# Investigating pain-related medication use and contribution to polypharmacy in adults with intellectual disabilities: a systematic review

**DOI:** 10.1186/s12916-024-03770-9

**Published:** 2024-12-02

**Authors:** Christine Pacitti, Deborah Cairns, Laura Ward, Barbara I. Nicholl

**Affiliations:** 1 School of Health & Wellbeing, University of Glasgow, Glasgow, G12 8TB UK; 2 Scottish Learning Disabilities Observatory, School of Health & Wellbeing, University of Glasgow, Glasgow, G12 8TB UK; 3 Health Informatics Centre, School of Medicine, University of Dundee, Dundee, DD1 9SY UK; 4 General Practice & Primary Care, School of Health & Wellbeing, University of Glasgow, Glasgow, G12 8TB UK

**Keywords:** Intellectual disability, Pain, Analgesia, Medications, Multimorbidity, Polypharmacy

## Abstract

**Background:**

Adults with intellectual disability experience more pain than adults without and, despite a higher number of medications being prescribed, may be less likely to receive medication for pain. We conducted a systematic review of existing literature on medication for pain and painful conditions in adults with intellectual disability to explore if there is any association with polypharmacy, multimorbidity or demographic characteristics.

**Methods:**

This systematic review followed PRISMA guidelines. Medline, Embase, PubMed, PsycINFO, Web of Science, CINAHL, Cochrane Library and Scopus were searched from January 2000 to 21st October 2024. We included original, peer-reviewed observational, qualitative or mixed-method studies published in English with data on medication for pain or painful conditions in adults with intellectual disability. Two independent reviewers performed study selection, data extraction, and quality assessment; disagreements were resolved by a third reviewer. Adapted Newcastle–Ottawa Scale or the Critical Appraisal Skills Programme for qualitative studies was used for quality assessment of included studies and findings were reported via narrative synthesis. PROSPERO registration: CRD42023415051.

**Results:**

Twenty-seven of 26,170 articles met the eligibility criteria. Adults with intellectual disability were more likely to have simple analgesic medication than non-steroidal anti-inflammatory drugs, opioids or adjuvant pain medications than the general population. Psychotropic medications were more commonly prescribed in adults with intellectual disability than medication for pain or painful conditions. Adults with intellectual disability and caregivers reported under-recognition and most likely under-treatment of pain.

**Conclusions:**

Adults with intellectual disability may receive less pharmacological management of pain with analgesics and medication for painful conditions despite the high prevalence of polypharmacy, suggesting pain is under-treated. Better assessment and pharmacological treatment of pain and painful conditions is a key future research priority to address this health inequality and improve quality of life for this vulnerable group of people.

**Supplementary Information:**

The online version contains supplementary material available at 10.1186/s12916-024-03770-9.

## Background

Intellectual Disability is a neurodevelopmental condition characterised by significant intellectual deficits present from birth, or which originate during the developmental period [1]. The estimated prevalence is 0.05–1.55% globally, depending on diagnostic criteria, health systems and data sources [2, 3]. Adults with intellectual disability experience poorer health and increased long-term health conditions (LTCs) at a younger age compared to the general population [4, 5]. These multiple LTCs are a contributing factor to poorer clinical outcomes and reduced quality of life in this population. Common health conditions experienced by people with intellectual disability include respiratory illness, poor oral health, gastrointestinal conditions, visual impairment, constipation, epilepsy, diabetes, cardiovascular problems and musculoskeletal pain [4, 6–9]. Dental infections, gastrointestinal conditions and musculoskeletal conditions are often painful. Adults with intellectual disability are already at an increased risk of pain associated with reduced physical activity, a higher rate of injuries and falls and reduced access to healthcare services [10–12]. Pain is defined as a multi-dimensional, subjective experience, categorised as either acute or chronic [13, 14]. Sudden onset, short-duration acute pain may have a protective role, either as a warning sign or to limit movement and prevent further injury and is generally associated with a cause, e.g. injury or illness [15, 16]. Chronic pain is persistent or recurrent pain lasting longer than 3 months and the cause may not be obvious [17]. The subjective nature of pain is a result of the complex interplay between multiple biological variables, psychosocial processes and previous pain experience; therefore, an individual’s experience of pain is unique to them [13]. The International Association for the Study of Pain (IASP) recognises pain occurs with at least as much, possibly more, frequency in individuals with significant cognitive and communication difficulties as the general population, and may not be easily recognized [18]. The previous misconception that adults with intellectual disability may have a higher pain threshold, is not supported by the evidence [18–20]. Pain assessment relies on self-report, in individuals with intellectual disability this may not be possible. The more severe the degree of intellectual disability, the greater the risk of pain being experienced but pain assessment is also more challenging [21]. This may be due to; communication difficulties, which may impact the ability to self-report, a reliance on proxy reporting (e.g. from family carers); challenges with conducting health assessments (e.g. due to behaviours that challenge [BtC]); and failure by health professionals to recognise and diagnose painful conditions where there may be atypical presentation [12, 22–24]. Under-recognition of pain may lead to delayed health care access, increased hospital admissions and preventable deaths [25].

In addition to increased pain risk, increased LTCs are associated with an increase in polypharmacy (use of multiple medications) [26]. Adults with intellectual disability are prescribed an average of three medications with polypharmacy risk increasing with severity of intellectual disability [27, 28]. Inappropriate polypharmacy increases the risk of adverse drug reactions, such as renal impairment with diuretics or constipation with anticholinergics or opiates, and it is known that adults with intellectual disability are more susceptible to adverse drug reactions in comparison to the general population [29, 30].

Chronic pain prevalence in adults with intellectual disability has been reported to be 15–18%, compared to 43.5% in the general population [31, 32]. The prevalence of chronic pain increases into older age in the general population, but less is known about chronic pain in the ageing intellectual disability population [33]. A Swedish registry-based study investigating pain and pain medication in older adults with intellectual disability (aged 55 + years), reported fewer analgesics and pain-related medications being prescribed compared to the general population [34]. Previous studies suggest that adults with intellectual disability have a higher pain threshold, however, these findings need to be interpreted with caution [19, 20]. Firstly, pain sensation and perception may be altered in some individuals with intellectual disability associated with a specific diagnosis [35–37], and secondly, pain in adults with intellectual disability is currently under-recognised and untreated [22].

The increased, and often complex, LTCs experienced by adults with intellectual disability indicate pain may be experienced across the lifespan at least as much, and possibly more than, the general population [3, 6, 38].

There is a significant knowledge gap on medication for pain or painful conditions in adults with intellectual disability. This systematic review aims to synthesise the available literature and examine.

1. What is known about the use of pain-related medication in relation to the types of analgesics used and do demographic factors or degree of intellectual disability influence this?
2. Where does pain-related medication sit in the context of multimorbidity and polypharmacy for adults with intellectual disability?
  - Are pain medications used for specific painful conditions or only where there is a suspicion of pain?
  - Is physical or mental illness associated with pain medication in adults with intellectual disability?
3. What are the views of adults with intellectual disability, caregivers and healthcare professionals on pain medication use?

## Methods

This mixed-methods systematic review was conducted in accordance with PRISMA guidelines [39], and the protocol was registered with the International Prospective Register of Systematic Reviews (PROSPERO), registration number CRD42023415051. Eight online bibliographic databases (Medline, Embase, PubMed, PsycINFO, Web of Science, CINAHL [Cumulated Index to Nursing and Allied Health Literature], Cochrane Library and Scopus) were searched on the 1st of March 2023 and updated on the 21st of October 2024, with a comprehensive search strategy (see Additional File 1) finalised with input from an information specialist librarian using terms for “intellectual disability” (including historical terms), “pain”, “analgesia” and “medications”. As summarised in Table 1**,** inclusion and exclusion criteria were defined according to the Population, Exposure, Comparator, Outcome, Study design (PECOS) framework. Included studies had to include information on pain medication or painful conditions and medication in adults with intellectual disability in all settings.

**Table 1:** Study criteria in accordance with the Population, Exposure, Comparator, Outcome (PECO) framework

Criteria	Inclusion	Exclusion
Population	• Adults with intellectual disability *OR* a diagnosis associated with intellectual disability, adults diagnosed with autistic spectrum disorder or cerebral palsy where ≥ 50% of the cohort had co-occurring intellectual disability • Participants ≥ 18 years or ≥ 50% of the cohort were adults and separate results reported • All community, residential and healthcare settings included	• Adults with Autistic Spectrum Disorder or Cerebral Palsy without co-occurring intellectual disability • Participants < 18 years of age
Exposure	• Any analgesic or non-analgesic medication used to manage pain • Any medication used to treat a painful condition	• Any management related to surgical interventions
Comparator	No comparator or control population essential but where a comparator group is included, the findings will be reported	
Outcomes	a) Information about the use of medication for pain and/or painful conditions in adults with intellectual disability and association with demographic factors or degree of intellectual disability b) Information about polypharmacy/multimorbidity and pain-related medication in adults with intellectual disability and if physical or mental health diagnosis influence how pain-related medication is used c) Information on the views of adults with intellectual disability, caregivers and health care providers on pain and related medication	
Study design	Peer-reviewed original, observational (including cross-sectional and longitudinal), qualitative or mixed methods. Studies published in English since 2000	Systematic reviews, conference articles, scoping reviews, letters and single case studies

### Study selection and data extraction

First author (CP) retrieved eligible studies from database searches for assessment against inclusion/exclusion criteria, and CP completed the initial title screen. Four independent reviewers double-screened titles and abstracts of identified articles for eligibility (CP and either DC, BN or LW). Full-text articles were screened independently by two reviewers (CP and BN). Disagreements between reviewers were resolved through discussion or by consultation with a third reviewer (DC). Reference lists of included studies were also scrutinised for any relevant articles. A data extraction form was developed to assess study design, setting, size, demographics, context, data source, inclusions/exclusions, method of analysis, outcomes and findings for all included studies.

### Analysis and quality appraisal

Results were synthesised narratively due to the diverse characteristics, methodologies and heterogeneity of included studies. Quality appraisal of included papers was conducted by two reviewers (CP and BN) using a modified version of the Newcastle–Ottawa Scale (NOS) for observational studies and CASP (Critical Appraisal Skills Programme) tool (2018) for qualitative studies (see Additional file 2: Table S1 and S2) [40, 41]. No studies were excluded on the basis of quality.

## Results

**Fig. 1:**
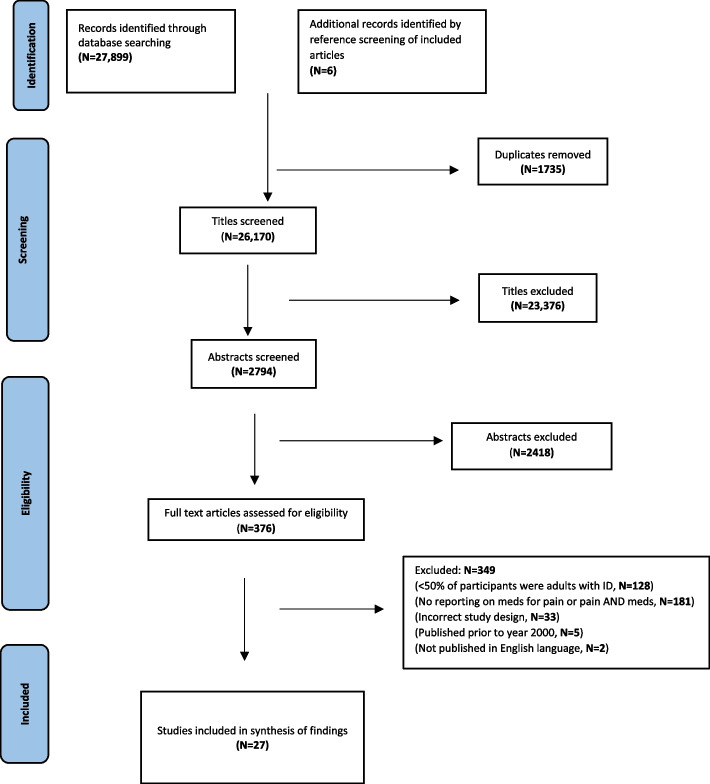
PRISMA flow diagram of article screening and selection process

A total of 27,899 references were retrieved from eight databases and an additional six articles were retrieved from reference reviews of relevant papers (Fig. 1). After duplicate removal, 26,170 titles were screened and 23,376 were excluded for not being relevant to the research question or population. Two reviewers independently examined 2794 abstracts and subsequently 376 full-text articles, with 27 meeting the inclusion criteria for the study.

### Characteristics of included studies

Table 2 summarises the characteristics of the 27 included studies. Twenty-three were quantitative, 14 were recorded linkage studies [31, 34, 42–53], two of which used the same register [34, 48], eight studies reported cross-sectional survey results of adults with an intellectual disability or caregiver/proxy, from specialist health and social care services [54–61] and one was a cohort study [62]. Four remaining studies were qualitative in design, one involved intellectual disability users of a specialist health service [63], two studies included carers of adults with intellectual disability [64, 65] and one included adults with intellectual disability and a carer [66] identified from service provider organisations. One observational study reported views of healthcare professionals [67] and two reported views of adults with intellectual disability [63], caregiver’s responses were reported in three studies [31, 42, 64].

**Table 2:** Characteristics of included studies

**Study** *(author, year)*	**Country**	**Purpose of study**	**Study design setting** **(comparison sample)**	**Data source and ascertainment of ID**	**ID cohort** *(N, sex, age)*	**Severity of ID and**	**ID aetiology**
Kerins et al. (2008)	USA	Medical conditions and meds common in DS	Retrospective study in statewide specialist OP clinic	Medical records	*Ν*—141, *F*—40%, mean—51 years*. *(31—65 years)	-	DS- 100%
Walsh et al. (2011)	Ireland	Factors associated with chronic pain in adults with ID	Cross-sectional survey in community setting	Psychology assessment. Carer questionnaire. National ID Database	*N*—753, *F*—42%, mean—42 years (+ / − 12)	Mild—26%, Mod.—42%, Sev.—22%	-
Turk et al. (2011)	England	Adults with ID health problem report compared to carer report	Primary care data analysis Adult with ID and carer interview	Adults with ID/carer interview. ID health/social care register	*N*—98, *F*—33%, mean—41 years (18–83 years)	-	-
Findlay et al. (2013)	England	Meaning attributed to acute and chronic pain by adults with ID	Qualitative study, semi-structured interviews. Community/nursing home	Adults with ID interview. Specialist health team register	*N*—15, *F*—53%, age—“early/mid/late” decade	Mild, mod.—100%	-
de Knegt et al. (2013)	Netherlands	Does format of facial/numeric pain scales affect pain understanding in adults with DS	Cross-sectional study of relationship between pain experience and cognitive function	Caregiver questionnaire. Dutch residential care centres	*N*—106, *F*—47%, mean—37 years (+ / − 11)	Mild—33%, Mod.—56%, Sev.—11%	DS- 100%
Sinnema et al. (2013)	Netherlands	Cause of hospitalisation, meds, anaesthetic issues, symptoms, and recovery in PWS	Cross-sectional study residential, family, or independent settings	PWS adult and caregiver questionnaire, PWS parent association. ID physicians. Medical records	*N*—97, *F*—52%, mean—36 years (+ / − 12)	Borderline—10%, mild—52%, Mod.—30%, Sev.—8%, *N*—97	PWS- 100%
Doan et al. (2013)	Australia	Demographic and medical characteristics linked to meds in primary health care	Cross-sectional data derived from RCT Community primary healthcare setting	CHAP trial data Participants/caregivers interview Medical records	*N*—117, *F*—39%, mean—33 years (19–71 years)	Mild—12%, Mod.—25%, Sev. prof.—31%, unknown = 32%	DS–13%
Findlay et al. (2014)	England	Caregivers’ response to possible/actual pain; pain recognition, and emotional impact on caregivers	Qualitative study in community and residential settings	Parent and professional caregiver interviews Carer groups and residential homes	*N*—11 (of caregivers of adults with ID) Age, gender NR	-	DS—18%
Cocks et al. (2016)	Australia	Health disparities in adults with ID compared with gPop	Cross-sectional study in community and residential settings (*N*—7182)	Adults with ID/proxy interviews Western Australia Disability Services Commission database	*N*—328, *F*—41%, mean—37 years (18–82 years)	-	DS—13%, CP—10%, other—10%
O’Dwyer et al. (2016)	Ireland	PP in adults with ID over 40 years	Cross-sectional study in community and residential settings	Adult with ID and/or proxy interview IDS-TILDA database	*N*—736, *F*—55%, mean—54 years (+ / − 8)	Mild—23.9%, Mod.—46.5%, Sev.—24.2%, Prof.—24.2%	-
Bowring et al. (2017)	Jersey	Med’s prevalence, focussing on psychotropic meds, in adults with ID	Cross-sectional study of adults with ID in community and residential settings	Carer interview Health and social care register. Education Dept. Record of Needs, service provider	*Ν*—265, *F*—49%, mean 41 years (18–85 years)	Mild—47%, Mod.—31%, Sev.—12%, Prof.—10%	DS—14%, FXS—0.75%, Soto syndrome—0.75%
Peklar et al. (2017)	Ireland	Meds patterns and associated MM in older adults with ID compared to matched gPop	Cross-sectional study in community dwellings (*N*—8081)	IDS-TILDA and TILDA	*N*—238, *F*—44%, age: 50–59 years—64%, 60–69 years—28%, > 70 years—8%	Mild—36%, Mod.—51%, Sev. prof.—13%	-
de Knegt et al. (2017)	Netherlands	Cognitive function and association with ability to affirm pain and describe pain intensity/effect in DS	Cross-sectional study in residential settings	Medical Records, Self-report Dutch DS Website	*N*—224, *F*—47%, age—38 years (+ / − 11)	Mild—25%, Mod.—66%, Sev.—9%	DS—100%
Axmon et al. (2017)	Sweden	Comparison of PIM in older adults with ID and matched gPop. Association of demographic effects with PIM	Register-based longitudinal study in community and residential settings between 2006 and 2012 (*N*—7936)	Swedish Total Population register, LSS and Prescribed Drug Registers	*N*—7936, *F*—45%, 2006 age = 49–90 years; 2012 age = 55–96 years	-	-
Salomon et al. (2018)	Australia	Compare meds advised by GPs for adults with ID versus non-ID	Cross-sectional study of primary care records (*N*—770,509)	BEACH GP dataset, GP assessment	*N*—482, *F*—49%, 25–44 years—32%, 45–64 years—31%, 65–74 years—4%, > 75 years—3%	-	-
Axmon et al. (2018)	Sweden	Pain/pain meds comparison in older adults with IDs with matched gPop	Cross-sectional register-based study in residential and community settings (*N*—7936)	Swedish national patient, LSS and Prescribed Drug Registers	*N*—7936, *F*—45%, mean—64 years (55–96)	-	-
Hove et al. (2018)	Norway	Compare rates of drug use in adults with ID and gPop in different age groups	Cross-sectional study in community settings (*N*—289,325)	Adults with ID questionnaire. Public Statistics databases. Norwegian Prescription Database	*N*—593, *F*—46%, mean—42 years (+ / − 14.5)	Mild—22%, Mod.—41%, Sev.—18%, Prof.—13%	-
Carfi et al. (2019)	Italy	Clinical characteristics of DS, chronic disease prevalence and meds	Cross-sectional study in specialist outpatient clinic	Medical records, physician assessment. Referral by DS associations and family physicians	*N*—421, *F*—51.1%, mean—38 years (+ / − 12.8)	-	DS—100%
Rosseau et al. (2019)	France	Examining meds, health status and medical devices in severe and complex disability	Cross-sectional study in specialist residential centres	Medical records Referring physician	*N*—474, *F*—46%, 18–34 years—46%, 35–49 years—32%, 50–68 years—22%	Prof- 100%	-
Segerlanz et al. (2019)	Sweden	Pain meds with a cancer diagnosis in older adults with ID compared to gPop	Cross-sectional registry-based study in community and residential settings (*N*—877)	Swedish National Patient, LSS, Prescribed Drug and Population registers. LSS register records	*N*—555, > 55 years Sex NR	-	-
Pickering et al. (2020)	France	Pain assessment and management for adults with ID	Cross-sectional study in specialist institutional care settings	Caregiver survey Medical records	*N*—218, *F*—44%, mean—47 years (34–81 years)	Sev.—100%	Congenital—51%, acquired—28%, congenital + acquired—6%, other—15%
McMahon et al. (2020)	Jersey	Prevalence of meds and factors associated with PP in adults with ID	Cross-sectional register-based study in residential and community settings	Adults with ID/proxy interview. Prescription charts. Med’s administration records Self-reported medication	*N*—217, *F*—44%, mean—45 years (+ / − 16)	Mild—50%, Mod.—26%, Sev.—15%, Prof.—9%	DS—13.4%
Holmes et al. (2021)	Australia	Describe and quantify pain in nonmobile adults with CP using NCAPC	Observational cohort study in a specialist transition clinic	Carer interview Medical record	*N*—15, sex NR, mean—19 years (18–33)	-	-
Drozd et al. (2021)	England	Adults with ID description of orthopaedic or trauma hospital experiences	Qualitative study semi-structured interviews in hospital setting	Adults with ID/carer interview, self-advocacy groups/national support organisations	*N*—5, *F*—3, mean—37 years (25–45, 1 age NR)	-	-
Guan et al. (2022)	Canada	Comparison of opioid-related adverse events, toxicity, OUD and dose increase. In adults with ID and gPop	Cross-sectional register-based study in community settings (*N*—76,708)	Health insurance database Medical records Narcotics database	*N*—19,814, *F*—43%, mean—37 years (+ / − 17)	-	-
Bernal- Celestino et al. (2022)	Spain	Carer perception of pain and use of analgesics, in adults with ID	Cross-sectional caregiver survey in community settings	Caregiver questionnaire survey in organisations supporting adults with ID	*N*—130, *F*—40%, mean—43 years (+ / − 14)	Mild—13%, Mod.—22%, Sev.—35%, Prof.—17%	-
Nieuwenhuijse et al. (2022)	Netherlands	Professional caregivers’ perception on good/poor QoL in adults with prof. ID	Qualitative study, semi-structured interviews in residential and day centre settings	Caregivers interview in organisations providing residential/day support	*N*—11 (caregivers of adults with ID) age or gender NR	Prof -100%	-

Studies were conducted in 13 high-income countries across three continents: Europe *N* = 21 [31, 34, 42, 45–48, 50–52, 55–61, 63–66], Australia *N* = 4 [43, 44, 49, 62] and North America *N* = 2 [53, 54]. Most studies were conducted in community or combined community and residential or nursing settings. Five were set in specialist residential facilities [56, 58, 59, 63, 67], three studies in specialist outpatient clinics [54, 55, 62] and one in a hospital in-patient setting [66].

The total number of participants across the 27 studies was 41,942 with a range from *N* = 5 to *N* = 19,814. Due to overlap between studies that drew samples from the same registers [34, 45–48, 51, 52] or settings [56, 58, 59, 67], it is not possible to give an accurate total of adults with intellectual disability included as participants. Two studies did not report on age, gender or residential status [51, 64].

Pain medication in adults with intellectual disability was the primary outcome in three studies [34, 51, 53]. Most studies investigated medication use [43, 45–50, 52] and comorbid health conditions [42, 44, 54, 55, 57, 59] in adults with intellectual disability and, if reported, pain medication was considered in regard to medication in general. Primary outcomes investigated in the remaining studies were pain experience [58, 63, 65, 66], pain assessment [56, 64, 67] and pain prevalence [31, 60, 62].

Severity and aetiology associated with intellectual disability were reported in eight studies [43, 44, 46, 52, 56–58, 67]. Four studies included only adults with Down syndrome (DS) [54, 55, 58, 68], one study only adults with Prader-Willi syndrome (PWS) [57] and one only adult with intellectual disability and Cerebral Palsy [62]. Four studies included participants with a specific severity of intellectual disability [59, 63, 65, 67]. Seven studies reported the severity of intellectual disability [31, 45, 50, 59, 60, 63, 65] and three reported findings on underlying aetiology associated with intellectual disability [54, 55, 64], nine studies did not report intellectual disability aetiology or severity [34, 42, 47–49, 51, 53, 62, 66].

Comparator samples were included in eight studies [34, 44, 47–51, 53], six of which matched with general population samples for age and sex [34, 47, 50, 51, 53, 69]. One comparator study reported underlying aetiology associated with intellectual disability [44] and two reported severity of intellectual disability [47, 50]. No comparator studies reported both aetiology and severity of intellectual disability.

### Pain

Table 3 summarises data on pain, medication for pain or painful conditions, comorbid health conditions and additional medication. Pain diagnosis, assessment or painful condition data was provided in twenty studies [31, 34, 42, 44, 47, 51, 53–60, 62–67]. Walsh et al. (2011) found older age was not associated with pain in adults with intellectual disability aged 20–85 years via caregiver survey [31]. In contrast, three more recent studies reported findings that older age was associated with pain [44, 55, 59]. However, Walsh et al. (2011) had a low response rate (31.6%), and the authors acknowledged that proxy reporting may not be an accurate method of pain measurement in adults with intellectual disability. Turk et al. (2012) found higher self-reported pain prevalence than caregivers’ proxy report of pain in adults with intellectual disability [42]. Common painful health conditions reported were associated with musculoskeletal, gastro-oesophageal reflux disease, constipation and respiratory conditions [44, 47, 54–56, 62]. Distress or dysregulated behaviour associated with pain prevalence was reported in three studies [31, 42, 59]. Living in institutional residential settings was associated with increased pain in two studies [31, 45].

**Table 3:** Pain, comorbid health conditions and medication in adults with intellectual disability

Study	Pain diagnosis	Pain measurement or assessment	Pain-related medication	Comorbid health conditions reported	Additional medications reported
Kerins et al. (2008)	Osteoporosis—24%, GORD—14%, arthritis—13%, IBD—6%, cancer—2%	-	GORD—20%, alendronic acid—22%, antispasmodics—3% “Other meds” included analgesia	From medical records: Dementia—75%, thyroid—40%, skin—26%, seizures—21%, sleep apnoea—19%, lung—18%, urinary incontinence—18%, cardiac—18%	From medical records: Anti-anxiety—16%, antidepressants—20%*,* antipsychotics—14%, AEDs—26%, anti-hypertensive—11%, cholesterol↓ agents—10%, cholinesterase inhibitors—10%, hormones—14%, hypothyroid—36%, respiratory meds—27%
Walsh et al. (2011)	chronic pain—15% mean 6.3 years. Pain more common in F, often ≥ 1 medical cause: Pain abdomen/legs—32%, lower back—25%, hips/pelvis—23%, head—20%	Verbal report/ facial expressions, irritability, crying, mood. 78% of participants experienced pain-related distress	Participants with identified pain—82.5% given pain meds (63% analgesic, 30% NSAID)	-	-
Turk et al (2011)	Head/stomach—41%, back—36%, leg/foot—29%, chest—27%, ear—25%, bottom—23%, throat—20% Pain in ≥ 1 area—70% Pain not reported—18%	-	Do participants use analgesics? Yes—61% Sometimes—11% No—27% Don’t know—1%	Medical record/participant report Eyes—40%, allergies—29%, weight/skin problems—23%, dental—20%, ears—19%, epilepsy/↑BP—16%, phobias—15%, depression—14%, BAD—13%, asthma—11%, anxiety—9%	From medical record—mean 1.7 meds per participant No meds—44%, From adults with ID report 2.4 meds per participant (*OTC meds included*) No medication—3%
Findlay et al (2013)	Current pain—53% Past pain—100% Pain in ≥ 1 category—87%	Pictorial aids, pain scales for adults with ID used	Pain medication—100%	-	-
De Knegt et al. (2013)	Skin—14%, Constipation, knee pain—6%, foot, back pain—5%, gout, inflamed gums, bowel disease—4%, Hip dysplasia—3%, hips, muscles, scoliosis, OA, eyes and GORD—2%	FAS better than NRS pain scale	Analgesic—5.6%	-	-
Sinnema et al. (2013)	Inguinal hernia—10%, erysipelas—5%, cholecystitis—3%, fractures—3%,	-	ATC A02—11.8%, A06—22.5%, A07—1%, D07—13.7%, M05—2.9%, N02—4.9%, R03—6.9%	-	From medical record: 0 meds—20%, 1 med—20%, 2–5 meds—45% ≥ 5 meds—15% Total ATC meds classes included: A—30%, B—12%, C—28%, G—27%, H—18%, J—7%, N (*not analgesic)*—87%, R (*not COPD)*—16%, S—5%
Doan et al. (2013)	-	-	Analgesic or NSAID—25% *(most common paracetamol)* GI medications—25% *(most common laxatives)*	-	From medical record: Psychotropics—35%, AEDs 26%, sex hormones/GU modulators—20%, topical steroid—16%, CV meds—11%, anti-asthma 9%, antihistamines 7%, anti-microbials 7%
Findlay et al. (2014)	-	-	10/11 caregivers tried medical solutions	-	-
Cocks et al. (2016)	Constipation—32%, arthritis—6%, Osteoporosis—4%, stomach ulcer—3%, cancer—2%. ↑arthritis.^a^	-	Pain relief—15% Meds for arthritis/ joint inflammation 5%	Participant report—*18–44 years*. ↑epilepsy, anxiety, cardiac issues, OCD, lung issue *(↓asthma)*, osteoporosis, stroke. ^a^ ≥ *45 years.*, ↑epilepsy, ↑cholesterol, lung issue *(↓asthma), less ↑BP* and* ↓* cancer^a^	Participant report↑ meds ^a^. ≥ 1 med—66%, ≥ 5 med—19%, ≥ 1 psychoactive med—25%, ≥ 1 AED—25% Meds from > 2 classes—36%
O'Dwyer et al. (2016)	Pain—47% GI conditions—30%, Joint disease—21%	-	ATC – A02B—24%, A06A—38%, M01A—10%, N02B—38% *(paracetamol*—*95%, paracetamol* + *codeine*—*6%, opioids*—*2%),* M05B—8%	Participant report: Eye—52%, mental health—48%, neurological—35%, endocrine—22% ↑ BP—15.2%, CV disease—12.1%	Participant report: ATC B01A—11%, HO3A—18%, N02A—43%, N03A—39%, N04A—16%, N05B—24%, NO5C—14%, N06C—26%, 14% N05C PP—32% Excessive PP—21.5%
Bowring et al. (2017)	-	-	ATC M—7.5%, N02A/B/C—21.5%, N03A—1.13%,	Medical record/participant report epilepsy—22%, depression—12%, schizophrenia—7%, Affective disorder—7%, psychotic condition—7%	Medical record/participant report Participants reporting meds—71% Psychotropic medication—37% ATC: A—31%, B—10%, C—15%, D—6%, G—11%, H—9%, J—6%, R—9%
Peklar et al. (2017)	↑Osteoporosis/arthritis, cancer, peptic ulcer and constipation ^a^	-	ATC ^a^ N02—3.1% (9th) v 3% (12th) M01—2.6% (12th) v 4.1% (7th) A06—4.4% (6th) v 0.5% (25th) A02—3.9% (8th) v 6.2% (8th) A03—1.8% (16th) v 0.5% (25th)	MM —73% v 50%, ↑BP—21% v 37%^a^ *(mm defined as* ≥ *2 chronic conditions)*	ATC meds—4.55 v 2.35^a^, A—3 × more meds, 17 x ↑ drug diversity, D—14 × more meds, 16 x ↑drug diversity, N—6 × more meds, 20 x ↑drug diversity, L—73% ↓ meds, ↑drug diversity, C—↓meds, ↑drug diversity Drug diversity – 95.8 v 7 *per 100 participants *^*a*^
de Knegt et al. (2017)	50% health conditions associated with pain	Self-reported pain using FAS and NRS	analgesics—5%	Participant report—sleep and/or depressive symptoms—7%	Participant report 7% with sleep/depressive disorder had hypnotic
Axmon et al. (2017	-	-	↓NSAIDs and tramadol ^a^	-	↑prescribing of PIMs ^a^
Salomon et al. (2018)	-	-	↑ Simple analgesics, ↓ NSAIDs and opioids, anti-ulcerant no difference ^a^	-	Medical record: ↑ Antipsychotics/anticonvulsants, antidepressant, anxiolytic no difference, ↓ hormones, cardiovascular, skin medications ^a^
Axmon et al. (2018)	MSK pain, visceral pain, urinary pain, headache, circulatory and respiratory pain	-	↑paracetamol and fentanyl, ↓NSAIDs/weak opioids. Overall ↓non-analgesic pain meds. (*TCAs, AEDs, SNRIs)* ^a^	EHR—↑ Epilepsy, pneumonia, type 2 diabetes^a^	-
Hove et al. (2018)	-	-	ATC M↓ in, analgesics comparable but ↓ in 41–60 years.^a^	-	Carer interview ATC —↑ A, N (*esp. Psychotropics, AEDs, Hypnotics, sedatives,*), R ↓ B, C, G,^a^
Carfi et al. (2019)	MSK disorders—23%, osteoporosis—20% (*age associated ↑).* GI diseases—8%, Psoriasis—4%, Atlanto-occipital dislocation—3%	-	Analgesics—1.43% (*age ↑),* GI meds—23%, PPI—8% *(↑with age* and* F)* Antigout meds—4% (*↑ in M),* Bone diseases – 14% *(↑ age, F),* Pain meds—1.4% – *(age ↑)*	Medical record: Visual impairment—73%, thyroid disease—50%, hypoacusis—27%, CHD – 25%, dementia—38.5% *(*> *40 years)* Epilepsy—3% 18–39 years, 13.3% > 40 years age,	Medical record: Mean meds—2.09 in 18–39 years. 2.77 in > 40 years. Total PP—10.5% [> *40 years.* —*15%, 18–39 years.*—*6.8%)*. Thyroid replacement—43.7%, Vit. D. —32.8%, Antidepressants—18.1%. Meds ↑ in > 40 years
Rosseau et al. (2019)	Pain—18–34 years—10%, 35–49 years—25%, 50–68 years—62%	Pain evaluation scale for patients with severe cerebral palsy	Analgesics/laxatives—age ↑ Antispasmodics, antidystonics, Osteoporosis prevention age ↓	Medical record: Epilepsy, orthopaedic, pulmonary, digestive illness, urinary problems, skin disorders. Co-morbid disorders ↓ with age except pressure fragility	Medical record—AEDs—75%, PP; *18–34 years*—*7.5 meds,* *35–49 years*—*8.3 meds,* *50–68 years*—*8.2 meds*
Segerlanz et al. (2019)	-	-	↓ pain med. ↑ paracetamol. ↓ NSAIDs/weak opioids. Strong opioids, AEDs, TCAs similar prevalence ^a^	-	EHR—↑anxiolytics and antidepressants ^a^
Pickering et al. (2020)	Pain diagnosed—55% Acute pain—48% (*trauma, inflammation, toothache),* neuropathic—9%	-	Participants pain identified— 98% received simple analgesic in previous month	-	Caregiver survey: Mean meds = 4, ≥ 5 meds—17% antipsychotics—66%, anxiolytics—44%, antidepressants—22%
McMahon et al. (2020)	-	-	ATC N02A/B/C—22% M, 28% F ↑ mild mod/↓ sev. prof A02, A06, D05, R03 include medication for painful conditions Analgesic = 9% of total medication	-	EHR ≥ 1 medication—85% ATC: A—23% a, D—11%, N—34% PP—38%, excessive PP-12%, 1 psychotropic—46% psychotropic PP—23%, antipsychotics 25%, ≥ 2 antipsychotics—3%
Holmes et al. (2021)	Spasticity, hip dysplasia, scoliosis	NCAPC	Baclofen—80%. gabapentin—33%, trihexyphenidyl—7%, bzds—47%	-	-
Drozd et al. (2021)	Orthopaedic trauma or surgery	-	Long wait or too little pain medication	-	-
Guan et al. (2022)	Cancer 5%, COPD 8%, RA 0.7%	-	Opioid prescribing/adverse events similar ^a^	Alcohol/substance misuse, hypertension similar. Diabetes slightly ↑ ^a^	BZD and stimulants similar ^a^
Bernal-Celestino et al. (2022)	Pain—22%, 88% of pain ≥ 1 area	-	Analgesics—26%, pain cohort—46% had analgesic *(pain cohort—37% communication barriers)*	Mean health conditions = 4 Epilepsy—41%, mental health—36%, sensory impairment—19%, thyroid—14%, skin problems—10%, ↑ cholesterol—9%	-
Nieuwenhuijse et al. (2022)	-	-	Caregivers may use analgesic to assess if pain affecting adult with PMID	-	-

### Medication for pain or painful conditions

Twelve studies [34, 43, 45–52, 55, 57] reported medication data using the Anatomical Therapeutic Chemical (ATC) classification system [70], while the remaining studies simply described medications reported. Simple analgesics (e.g. paracetamol, ibuprofen) were more likely than non-steroidal anti-inflammatory drugs (NSAIDs) (e.g. diclofenac, naproxen), opioids and non-analgesic medications (e.g. anti-depressants, anti-epileptic drugs [AEDS]) to be reported for adults with intellectual disability for pain treatment [31, 34, 43–47, 49–52, 55–57, 59, 67]. Similarly, in comparator studies, NSAIDs, opioids and non-analgesic medications were recorded less in adults with intellectual disability compared to the general population [34, 45, 47–51]. Common medications reported for painful conditions included laxatives, treatments for gastro-oesophageal reflux disease and musculoskeletal conditions [31, 43–45, 47, 52, 54, 55, 57, 59], consistent with common painful health conditions reported [44, 47, 54–56, 62]. Two studies reported that for those living in institutional residential settings, there was an increase in pain medication in comparison to individuals with intellectual disability living in the community [31, 45]. Where rankings were reported [43, 44, 47, 49, 50, 52, 55], the prevalence of analgesics ranked behind psychotropic medications in all but one study [52].

### Comorbid health conditions

Common LTCs reported, aside from painful conditions, were seizure disorders, sensory impairment, cardiovascular disorders, diabetes, depression and anxiety [34, 42, 44–47, 53–55, 59, 60, 68]. Four studies found adults with intellectual disability had more comorbid health conditions than adults without intellectual disability [34, 44, 47, 53]. An increase in LTCs in older adults compared to younger adults with intellectual disability was also reported [54, 55]. However, this contrasted with one study that found less comorbid health conditions in older age adults with intellectual disability [59].

### Additional medication

Twelve studies provided data for physical and mental health medication [43–47, 49, 50, 52, 54, 55, 57, 59], with data on mental health medication only given in four studies [48, 51, 53, 67]. A high rate of polypharmacy and psychotropic polypharmacy (the concurrent use of two or more psychotropic agents in one individual [71, 72]) was evident in adults with intellectual disability and was observed to be higher than in the general population [43–47, 52, 57, 59, 67]. Polypharmacy also increased in older adults with intellectual disability compared to younger adults with intellectual disability [47, 55, 59]. Excessive polypharmacy (10 or more medications [26]) was found in three studies [45, 47, 52]. The prevalence of pain was associated with polypharmacy and excessive polypharmacy in one study [45]. Psychotropic medication was the most common additional medication class and included anti-psychotics, anti-depressants, anxiolytics and anti-epileptic drugs [34, 43–47, 49–52, 54, 55, 57, 59, 67]. The presence of behaviours that challenge was associated with the increased use of psychotropic medication in adults with intellectual disability in two studies [43, 46].

#### Views of adults with intellectual disability, caregivers and healthcare professionals

Table 4 summarises the views of adults with intellectual disability (*N* = 15), caregivers (*N* = 823) and healthcare professionals (*N* = 307) on pain medication where reported. Reviewed literature shows that pain is under-recognised, and where analgesia is used, adults with intellectual disability report that it is not always helpful [42, 63]. Caregivers used analgesia if they thought the person they supported experienced pain. Caregivers acknowledged and expressed concern on the challenge of recognising pain in adults with intellectual disability in order to treat it [64]. There was a disparity between pain prevalence reported by experienced caregivers for the adults with intellectual disability they know well, and pain reported by the adults with intellectual disability themselves [31, 42, 64].

**Table 4:** Studies including data from adults with intellectual disability, caregivers and healthcare professionals

**Reference** Author (year)	**Views of adults with intellectual disability**	**Views of caregivers of adults with intellectual disability**	**Views of healthcare professionals**
Walsh et al. (2011)	Not investigated	Adults with intellectual disability used verbal report, facial expressions, grimacing, irritable mood, crying, mood change to express pain. Agreed prescription pain meds were adequate, over half agreed over-the-counter medication effective. Most satisfied with doctor treating adults with intellectual disability, but over half observed adults with intellectual disability were not satisfied with doctor, half of doctors’ base pain assessment on caregiver report.	Not investigated
Turk et al. (2011)	Not investigated	According to carers, 95% of adults with intellectual disability could express pain and 49% experienced it	Not investigated
Findlay et al. (2013)	Pain described with negative meaning, strong imagery, various causes suggested, little reported about coping with pain. Variable reporting to carers/hiding pain. Held belief that others can tell if someone is in pain	Not investigated	Not investigated
Findlay et al. (2014)	Not investigated	“Art” to detect pain, observing how adults with intellectual disability expressed pain. Recognising/treating pain is complex and ambiguous. Some described negative emotional impact/dissatisfaction with pain assessment/management by healthcare professionals	Not investigated
Pickering et al. (2020)	Not investigated	Not investigated	92% aware of pain assessment methods. No knowledge on using them, would like training. No views reported on pain medication

### Study quality

The scoring for each quality assessment can be found in Additional File 2. All four qualitative studies had high CASP scores. Using NOS, 15 studies were judged to be of fair or good quality [31, 42–47, 52, 54–56, 58, 59, 62–67] and eight of poor quality. This was primarily due to (1) a lack of detailed information on response rate and non-responders and/or (2) a lack of comparability between sub-groups [34, 48–51, 53, 60]. However, this is likely because of a lack of data on the aetiology and severity of intellectual disability rather than an inherent weakness in the study design.

## Discussion

This systematic literature review is the first to present a comprehensive synthesis of evidence on the use of pain-related medication in adults with intellectual disability. The findings demonstrate adults with intellectual disability may not be receiving appropriate pharmacological pain management or treatment of painful conditions, which may contribute to unmet health needs. Included studies that reported on pain-related medication for both the general adult population and adults with intellectual disability indicate a lower prevalence of pain medication, i.e. analgesics and adjuvant pain medications, for adults with intellectual disability [34, 47–51]. Twenty studies reported less pain-related medications than expected given the amount or type of pain and/or painful LTCs reported in adults with intellectual disability [31, 34, 42–52, 54–57, 60, 66, 68]. However, there was much variability between and in the quality of included studies by methodological approach, study populations, settings, medication classification systems used, and health conditions studied. The most common pain medications used for adults with intellectual disability were paracetamol and ibuprofen [31, 34, 43, 45, 49, 51, 56, 57, 67]. This may be, in part, due to healthcare professionals trying to manage polypharmacy or adverse effects such as exacerbation of underlying gastrointestinal disorders commonly seen in this population [27, 73, 74]. NSAIDs, opioids and adjuvant pain medications (e.g. anti-depressants, anti-epileptic drugs) are used less in adults with intellectual disability than the general population comparator groups [34, 44, 45, 47–51]. There was reduced medication for pain or painful conditions with increasing severity of intellectual disability, despite the potential increased risk of pain experienced by people with profound intellectual and multiple disability (PIMD) [8, 75–77]. This increased risk is associated with reduced mobility, unmet postural care needs, dysphagia, spasticity, gastro-oesophageal reflux disease and musculoskeletal disorders [75, 77, 78]. Walsh et al. (2011) reported severity of intellectual disability was not associated with pain [31], however, they did not include any adults with profound intellectual disability. Axmon et al. (2018) suggested that there are no biological or physiological reasons why pain prevalence would differ among people with or without intellectual disability, and differences in pain diagnoses are caused by other factors related to the individual, the caregivers and the health care system [34]. Walsh et al. (2011) and Axmon et al. (2018) did not include data on the cause or severity of intellectual disability for participants. Both studies conflict with findings from Robertson et al. (2018) that individuals with severe to profound intellectual disability are more likely to have associated medical conditions, and therefore increased medications, and potential pain, than those with mild to moderate intellectual disability [79, 80]. This may demonstrate a further disparity in pain care associated with increasing severity of intellectual disability and known barriers to pain diagnosis [34]. However, specific research on the incidence and treatment of pain in adults with profound and multiple intellectual disabilities would give a clearer picture. There are genetic diagnoses associated with intellectual disability, such as Down syndrome with an increased risk of musculoskeletal disorders and, Williams syndrome with a high prevalence of gastro-intestinal conditions [81–83]. Where studies exclude specific severities of intellectual disability or focus on a particular associated aetiology, there is a risk that the study sample may not be representative of the wider population. For example, adults with a diagnosis of Down syndrome experience a higher prevalence of thyroid disorders and Alzheimer’s dementia at an earlier age than adults without Down syndrome [84, 85]. There is a clear chasm in our knowledge on pain, and therefore pain treatment, associated with intellectual disability aetiology and severity.

Increased pain medication in older adults with intellectual disability and for those living in residential institutional settings was observed, and findings suggest healthcare professionals would welcome training on using pain assessment methods tailored to adults with intellectual disability [31, 44, 45, 55, 59]. The current review found that medication used by adults with intellectual disability did not appear to correlate with the diagnoses reported [42, 44–47, 54, 55]. This resonates with findings from Ho et al. (2021) who found over half of adults with intellectual disability and elevated cardiovascular disease risk, were not prescribed statin therapy recommended in cardiovascular disease risk reduction guidelines [86, 87]. Similarly, Weise et al. (2017) reported adults with intellectual disability were significantly less likely to be prescribed medications or advised to take over-the-counter medications after a primary care GP consultation, compared to adults without intellectual disability [88]. Adults with intellectual disability had more LTCs than the general population [34, 44, 47, 53] which is widely acknowledged in the intellectual disability literature [4, 85]. Increased comorbid health conditions were observed in older adults with intellectual disability compared to younger adults [54, 55], similar to the general population [89]. Rosseau et al. (2019) reported conflicting results with less comorbid health conditions in older adults with intellectual disability [59]. However, they only included adults with profound and multiple intellectual disabilities, with the majority (78%) aged between 18 and 49 years and only 22% aged between 50 and 68 years; it is therefore not a representative sample of the wider intellectual disability population. Existing studies in community-dwelling older adults, report female sex, living alone, poor self-rated health and excessive polypharmacy are associated with analgesic use, but these studies did not include adults with intellectual disability [90–92]. Tansug et al. (2021) reported nursing home residents experienced more pain than community-dwelling older adults, and Bauer et al. (2016) reported that pain may be under-treated in older adult residential settings, particularly in people with cognitive decline with impaired verbal communication [93, 94]. Neither Tansung et al. (2021) nor Bauer et al. (2016) included people with intellectual disability. Research is required to determine if similar factors influence pain medication in adults with intellectual disability. Increased exposure to polypharmacy and psychotropic polypharmacy was observed, particularly anti-psychotics, consistent with previous findings of over-prescribing of psychotropic medication [95]. Increased severity of intellectual disability was associated with increased polypharmacy and excessive polypharmacy [45, 52]. One study reported increased pain prevalence was associated with polypharmacy; however, pain medication was less frequently reported than psychotropic medications overall, suggesting medication for pain or painful conditions is under-prescribed, in contrast with psychotropic medication [43–46, 51]. Findings in this review are consistent with established knowledge on psychotropic over-prescribing, at higher doses, in adults with intellectual disability, where the prescribing of psychotropic drugs is greater than the recorded rate of psychiatric diagnosis [46, 52, 95, 96].

Between 10 and 15% of adults with intellectual disability experience behaviours that challenge. Behaviours that challenge describe behaviours, e.g. self-injury, aggression, or destruction of property, that present difficulties to individuals, caregivers, and health-care services [97, 98]. Physical or psychological discomfort contributes to behaviours that challenge in adults with intellectual disability and has been associated with pain-related discomfort in headache, ear infection, and gastro-intestinal discomfort [99, 100]. Anti-psychotics are commonly prescribed to manage these behaviours, despite a lack of evidence of benefit [46, 101]. Exploring if medication for pain, or suspected painful conditions, in place of e.g. anti-psychotics, may be a potential strategy to reduce risks of unnecessary psychotropic medication prescribing and improve quality of life for some individuals where behaviours that challenge are related to pain discomfort [99, 102]. Carr & Owen-DeSchryver (2007) observed both behaviours that challenge and pain were improved, when medications to reduce pain and discomfort for ear infection, colds and flus involving fever, constipation and injury were administered [99]. However, benzodiazepines, antidepressants and anti-epileptic drugs can also be used to manage pain or painful conditions [103–105]. Due to inconsistent diagnostic data, and variable methodology of included studies, it cannot be assumed these three classes of psychotropic medication are being used for mood or behaviour that challenges.

### Strengths

This is the first study to synthesise data investigating pain-related medication and contribution to polypharmacy in adults with intellectual disability. This review benefits from a comprehensive and robust search strategy following rigorous procedures. Broad inclusion criteria allowed us to capture all relevant literature on medication for pain or painful conditions, demonstrating inequalities in the pharmacological management of pain with analgesics and medication for painful conditions in adults with intellectual disability.

### Limitations

Included studies reported findings for adults with intellectual disability (18 years of age and older). As a result, some studies of interest to the topic were excluded because they included a small number of participants under 18 years of age but did not report this group separately [24, 106, 107]. Only four included studies had the primary outcome of interest as medication for pain in adults with intellectual disability [34, 51, 60, 67]. Of these, two used similar older adult registry-based population samples [34, 51] and there was variability in how medication was classified and reported. Outcomes of interest for this review were not the focus of the majority of included studies. There were no data in any studies on medication purchased over the counter—simple analgesia is commonly available in most retailers—and it is not known how often adults with intellectual disability or caregivers purchase these medications. Despite access to pain management being a fundamental human right [108], the current evidence extends the picture of overall health inequality that adults with intellectual disability continue to experience globally [109]. Furthermore, the studies included were all from high-income countries in Europe, North America and Australia. There were no studies that met inclusion criteria from low- and middle-income countries, despite the higher prevalence rate of intellectual disability, examining medication for pain and painful conditions in this vulnerable group [110]. Such research is necessary to better inform the global picture of pain care in adults with intellectual disability. The adapted NOS used for quality assessment has also not been validated. However, our edits only optimised the wording to include intellectual disability and correspond with the included study designs and did not alter the criteria assessed.

### Implications for research

This review highlights the need for further specific research on the pharmacological treatment of pain and painful physical health conditions in adults with intellectual disability, with more representative populations from all settings. There are also knowledge gaps in pain prevalence and determinants of pain risk in increased severity of intellectual disability and with specific genetic disorders. Guidance on improved prescribing for pain or painful conditions may help meet pain care needs of adults with PIMD who experience a greater risk of complex health conditions and pain.

### Implications for education

Increased awareness of pain assessment tools for adults with intellectual disability, and the role behaviours that challenge may have in pain communication, for health and social care training may better inform health professionals and caregivers on recognising pain in adults with intellectual disabilities.

### Implications for policy and practice

Further research on identifying barriers and enablers to assessment and treatment in adults with intellectual disability, particularly alongside other long-term health conditions (multimorbidity), could support future guidelines and decision-making on pharmacological pain management and appropriate treatment plans for painful health conditions. This may help to address pain care inequality in primary and secondary health care settings in this vulnerable population.

## Conclusions

This study suggests inequalities in the pharmacological management of pain and painful conditions in adults with intellectual disability. Future research on better pain prevention, assessment, and pharmacological treatment of pain and painful conditions with representative populations of adults with intellectual disability is necessary to strengthen the evidence on this topic. Pain undoubtedly impacts on the health and wellbeing of adults with intellectual disability, and improving pain care will help to improve the quality of lives of adults with intellectual disability.

## Supplementary Information

Additional file 1. Search terms examples.

Additional file 2. Quality assessment of studies.

## Data Availability

Not applicable. No datasets were generated or analysed during the current study.

## Abbreviations

ADRs: Adverse drug reactions
AED: Antiepileptic drug
ATC: Anatomical therapeutic class
BAD: Bipolar affective disorder
BEACH: Bettering the Evaluation & Care of Health
BP: Blood pressure
BZDs: Benzodiazepines
CHAP: Comprehensive Health Assessment Programme
CHD: Coronary heart disease
CNS: Central nervous system
COPD: Chronic obstructive pulmonary disease
CP: Cerebral palsy
CV: Cardiovascular disease
DS: Down syndrome
FAS: Facial affective scale
FXS: Fragile-X syndrome
GI: Gastrointestinal
GORD: Gastro-oesophageal reflux disease
GU: Genito-urinary
gPop: General population
IBD: Inflammatory bowel disease
IDS-TILDA: Intellectual Disability Supplement to the Irish Longitudinal Study on Ageing
LTCs: Long-term conditions
LSS: Act Concerning Support and Service for Persons with Certain Functional Impairments
Med(s): Medication(s)
MM: Multimorbidity
MSK: Musculoskeletal
NCAPC: Non-communicating adult pain checklist
NR: Not reported
NRS: Numerical rating scale
NSAIDs: Non-steroidal anti-inflammatory drugs
OA: Osteoarthritis
OCD: Obsessive-compulsive disorder
OP: Outpatient clinic
OTC: Over the counter
OUD: Opioid use disorder
PECOS: Population, Exposure, Comparator, Outcome, Study design
PIM: Potentially inappropriate medication
PIMD: Profound intellectual and multiple disability
PP: Polypharmacy
PPI: Proton pump inhibitor
PWS: Prader-Willi syndrome
QoL: Quality of life
RA: Rheumatoid arthritis
SNRI: Serotonin-noradrenaline reuptake inhibitor
TCA: Tricyclic antidepressant

## References

[1] International Association for the Scientific Study of Intellectual and Developmental Disabilities. [https://iassidd.org/about]

[2] McKenzie K, Milton M, Smith G, Ouellette-Kuntz H. Systematic Review of the Prevalence and Incidence of Intellectual Disabilities: Current Trends and Issues. Curr Dev Disord Rep. 2016;3(2):104–15.

[3] Perera B, Audi S, Solomou S, Courtenay K, Ramsay H. Mental and physical health conditions in people with intellectual disabilities: Comparing local and national data. Br J Learn Disabil. 2020;48(1):19–27.

[4] Cooper S-A, McLean G, Guthrie B, McConnachie A, Mercer S, Sullivan F, Morrison J. Multiple physical and mental health comorbidity in adults with intellectual disabilities: population-based cross-sectional analysis. BMC Fam Pract. 2015;16(1):110. 26310664 10.1186/s12875-015-0329-3 PMC4551707

[5] McMahon M, Hatton C. A comparison of the prevalence of health problems among adults with and without intellectual disability: A total administrative population study. J Appl Res Intellect Disabil. 2021;34(1):316–25. 32734651 10.1111/jar.12785

[6] Kinnear D, Morrison J, Allan L, Henderson A, Smiley E, Cooper SA. Prevalence of physical conditions and multimorbidity in a cohort of adults with intellectual disabilities with and without Down syndrome: cross-sectional study. BMJ Open. 2018;8(2): e018292. 29431619 10.1136/bmjopen-2017-018292 PMC5829598

[7] Hodapp RM, Core RE, Burke MM, Mello MP, Urbano RC: Chapter Six - Health issues across adulthood in Down syndrome. In: *International Review of Research in Developmental Disabilities. Volume 57*, edn. Edited by Hodapp RM, Fidler DJ: Academic Press; 2019: 229–265.

[8] van Timmeren EA, Waninge A, van Schrojenstein Lantman-de HMJ, van der Putten AAJ, van der Schans CP. Patterns of multimorbidity in people with severe or profound intellectual and motor disabilities. Res Dev Disabil. 2017;67:28–33. 28622657 10.1016/j.ridd.2017.05.002

[9] de Veer AJE, Bos JT. Boer RCN-d, Böhmer CJM, Francke AL: Symptoms of gastroesophageal reflux disease in severely mentally retarded people: a systematic review. BMC Gastroenterol. 2008;8(1):23. 18547405 10.1186/1471-230X-8-23 PMC2435531

[10] Robertson J, Emerson E, Gregory N, Hatto C, Turner S, Kessissoglou S, Hallam A. Lifestyle related risk factors for poor health in residential settings for people with intellectual disabilities. Res Dev Disabil. 2000;21(6):469–86. 11153830 10.1016/s0891-4222(00)00053-6

[11] Finlayson J, Morrison J, Jackson A, Mantry D, Cooper SA: Injuries, falls and accidents among adults with intellectual disabilities. Prospective cohort study. *J Intellect Disabil Res* 2010, 54(11):966–980. 10.1111/j.1365-2788.2010.01319.x 21040056

[12] Doherty AJ, Atherton H, Boland P, Hastings R, Hives L, Hood K, James-Jenkinson L, Leavey R, Randell E, Reed J *et al*: Barriers and facilitators to primary health care for people with intellectual disabilities and/or autism: an integrative review. *BJGP Open* 2020, 4(3). 10.3399/bjgpopen20X101030 PMC7465578 32605913

[13] Love-Jones SJ: Pain as a Subjective, Multidimensional Experience. In: *Pain: A Review Guide.* edn. Edited by Abd-Elsayed A. Cham: Springer International Publishing; 2019: 141–144.

[14] Raja SN, Carr DB, Cohen M, Finnerup NB, Flor H, Gibson S, Keefe FJ, Mogil JS, Ringkamp M, Sluka KA, et al. The revised International Association for the Study of Pain definition of pain: concepts, challenges, and compromises. Pain. 2020;161(9):1976–82. 32694387 10.1097/j.pain.0000000000001939 PMC7680716

[15] Hussien E, Hay D. Management of acute pain. Surg Infect (Larchmt). 2022;40(6):378–85.

[16] Bonezzi C, Fornasari D, Cricelli C, Magni A, Ventriglia G. Not All Pain is Created Equal: Basic Definitions and Diagnostic Work-Up. Pain Ther. 2020;9(Suppl 1):1–15. 33315206 10.1007/s40122-020-00217-w PMC7736598

[17] Treede R-D, Rief W, Barke A, Aziz Q, Bennett MI, Benoliel R, Cohen M, Evers S, Finnerup NB, First MB, et al. A classification of chronic pain for ICD-11. Pain. 2015;156(6):1003–7. 25844555 10.1097/j.pain.0000000000000160 PMC4450869

[18] Pain in Individuals with an Intellectual Disability: Scope of the Problem and Assessment Challenges [https://www.iasp-pain.org/resources/fact-sheets/pain-in-individuals-with-an-intellectual-disability-scope-of-the-problem-and-assessment-challenges/]

[19] Beacroft M, Dodd K. ORIGINAL ARTICLE: Pain in people with learning disabilities in residential settings – the need for change. Br J Learn Disabil. 2010;38(3):201–9.

[20] Doody O, Bailey ME. Understanding pain physiology and its application to person with intellectual disability. J Intellect Disabil. 2017;23(1):5–18. 28502222 10.1177/1744629517708680

[21] Defrin R, Lotan M, Pick CG. The evaluation of acute pain in individuals with cognitive impairment: a differential effect of the level of impairment. Pain. 2006;124(3):312–20. 16781070 10.1016/j.pain.2006.04.031

[22] Barney CC, Andersen RD, Defrin R, Genik LM, McGuire BE, Symons FJ. Challenges in pain assessment and management among individuals with intellectual and developmental disabilities. Pain Rep. 2020;5(4): e821. 32656458 10.1097/PR9.0000000000000822 PMC7302581

[23] Weissman-Fogel I, Roth A, Natan-Raav K, Lotan M. Pain experience of adults with intellectual disabilities–caregiver reports. J Intellect Disabil Res. 2015;59(10):914–24. 25827612 10.1111/jir.12194

[24] McGuire BE, Daly P, Smyth F. Chronic pain in people with an intellectual disability: under-recognised and under-treated? J Intellect Disabil Res. 2010;54(3):240–5. 20387264 10.1111/j.1365-2788.2010.01254.x

[25] White AS, R; Ding, J; Roberts, C; Magill, N; Keagan-Bull, R; Carter, B; Ruane, M;, Xiang XC, U; Tuffrey-Wijne, I; Strydom, A;: LeDeR Learning from lives and deaths-People with a learning disability and autistic people. In*.*: The Institute of Psychiatry, Psychology and Neuroscience (IoPPN) King's College, London; 2021.

[26] Khezrian M, McNeil CJ, Murray AD, Myint PK. An overview of prevalence, determinants and health outcomes of polypharmacy. Therapeutic Advances in Drug Safety. 2020;11:2042098620933741. 32587680 10.1177/2042098620933741 PMC7294476

[27] Haider SI, Ansari Z, Vaughan L, Matters H, Emerson E. Prevalence and factors associated with polypharmacy in Victorian adults with intellectual disability. Res Dev Disabil. 2014;35(11):3071–80. 25129201 10.1016/j.ridd.2014.07.060

[28] Mair A. ea: Scottish Government Polypharmacy Model of Care Group. Polypharmacy Guidance, Realistic Prescribing 3rd Edition, 2018. Scottish Government. In*.*; 2018.

[29] Erickson SR, Kamdar N, Wu C-H. Adverse Medication Events Related to Hospitalization in the United States: A Comparison Between Adults With Intellectual and Developmental Disabilities and Those Without. Am J Intellect Dev Disabil. 2020;125(1):37–48. 31877264 10.1352/1944-7558-125.1.37

[30] Osanlou R, Walker L, Hughes DA, Burnside G, Pirmohamed M. Adverse drug reactions, multimorbidity and polypharmacy: a prospective analysis of 1 month of medical admissions. BMJ Open. 2022;12(7): e055551. 35788071 10.1136/bmjopen-2021-055551 PMC9255409

[31] Walsh M, Morrison TG, McGuire BE. Chronic pain in adults with an intellectual disability: prevalence, impact, and health service use based on caregiver report. Pain. 2011;152(9):1951–7. 21497999 10.1016/j.pain.2011.02.031

[32] McGuire BE, Kennedy S. Pain in people with an intellectual disability. Curr Opin Psychiatry. 2013;26(3):270–5. 23508000 10.1097/YCO.0b013e32835fd74c

[33] Fayaz A, Croft P, Langford RM, Donaldson LJ, Jones GT. Prevalence of chronic pain in the UK: a systematic review and meta-analysis of population studies. BMJ Open. 2016;6(6): e010364. 27324708 10.1136/bmjopen-2015-010364 PMC4932255

[34] Axmon A, Ahlström G, Westergren H: Pain and Pain Medication among Older People with Intellectual Disabilities in Comparison with the General Population. *Healthcare (Basel)* 2018, 6(2). 10.3390/healthcare6020067 PMC6023323 29914061

[35] Cooley Coleman JA, Sarasua SM, Moore HW, Boccuto L, Cowan CW, Skinner SA, DeLuca JM. Clinical findings from the landmark MEF2C-related disorders natural history study. Mol Genet Genomic Med. 2022;10(6): e1919. 35416405 10.1002/mgg3.1919 PMC9184670

[36] Priano L, Miscio G, Grugni G, Milano E, Baudo S, Sellitti L, Picconi R, Mauro A. On the origin of sensory impairment and altered pain perception in Prader-Willi syndrome: a neurophysiological study. Eur J Pain. 2009;13(8):829–35. 18986815 10.1016/j.ejpain.2008.09.011

[37] McGuire BE, Defrin R. Pain perception in people with Down syndrome: a synthesis of clinical and experimental research. Front Behav Neurosci. 2015;9:194. 26283936 10.3389/fnbeh.2015.00194 PMC4519755

[38] Liao P, Vajdic C, Trollor J, Reppermund S. Prevalence and incidence of physical health conditions in people with intellectual disability - a systematic review. PLoS ONE. 2021;16(8): e0256294. 34428249 10.1371/journal.pone.0256294 PMC8384165

[39] Page MJ, McKenzie JE, Bossuyt PM, Boutron I, Hoffmann TC, Mulrow CD, Shamseer L, Tetzlaff JM, Akl EA, Brennan SE, et al. The PRISMA 2020 statement: an updated guideline for reporting systematic reviews. BMJ. 2021;372: n71. 33782057 10.1136/bmj.n71 PMC8005924

[40] CASP Qualitative Checklist [online]

[41] The Newcastle-Ottawa Scale (NOS) for assessing the quality of nonrandomised studies in meta-analyses

[42] Turk V, Khattran S, Kerry S, Corney R, Painter K. Reporting of health problems and pain by adults with an intellectual disability and by their carers. J Appl Res Intellect Disabil. 2012;25(2):155–65. 22473967 10.1111/j.1468-3148.2011.00642.x

[43] Doan TN, Lennox NG, Taylor-Gomez M, Ware RS. Medication use among Australian adults with intellectual disability in primary healthcare settings: A cross-sectional study. J Intellect Dev Disabil. 2013;38(2):177–81. 23550741 10.3109/13668250.2013.778968

[44] Cocks E, Thomson A, Thoresen S, Parsons R, Rosenwax L. Health status and use of medications by adults with intellectual disability in Western Australia. J Intellect Dev Disabil. 2016;41(2):87–96.

[45] O’Dwyer M, Peklar J, McCallion P, McCarron M, Henman MC. Factors associated with polypharmacy and excessive polypharmacy in older people with intellectual disability differ from the general population: a cross-sectional observational nationwide study. BMJ Open. 2016;6(4): e010505. 27044582 10.1136/bmjopen-2015-010505 PMC4823458

[46] Bowring DL, Totsika V, Hastings RP, Toogood S, McMahon M: Prevalence of psychotropic medication use and association with challenging behaviour in adults with an intellectual disability. A total population study. *J Intellect Disabil Res* 2017, **61**(6):604–617. 10.1111/jir.12359 28090687

[47] Peklar J, Kos M, O’Dwyer M, McCarron M, McCallion P, Kenny RA, Henman MC. Medication and supplement use in older people with and without intellectual disability: An observational, cross-sectional study. PLoS ONE. 2017;12(9): e0184390. 28877256 10.1371/journal.pone.0184390 PMC5587307

[48] Axmon A, Sandberg M, Ahlström G, Midlöv P. Prescription of potentially inappropriate medications among older people with intellectual disability: a register study. BMC Pharmacol Toxicol. 2017;18(1):68. 29070067 10.1186/s40360-017-0174-1 PMC5657112

[49] Carmela S, Helena B, Allan P, Julian T: Primary care for people with an intellectual disability — what is prescribed? An analysis of medication recommendations from the BEACH dataset. *BJGP Open* 2018, **2**(2):bjgpopen18X101541. 10.3399/bjgpopen18X101541 PMC6184100 30564718

[50] Hove O, Biringer E, Havik OE, Assmus J, Braatveit KJ, Holm SEH, Hermann M. Prevalence of drug use among adults with intellectual disabilities compared with drug use in the general population. Pharmacoepidemiol Drug Saf. 2019;28(3):337–44. 30747466 10.1002/pds.4741

[51] Segerlantz M, Axmon A, Gagnemo Persson R, Brun E, Ahlström G. Prescription of pain medication among older cancer patients with and without an intellectual disability: a national register study. BMC Cancer. 2019;19(1):1040. 31684896 10.1186/s12885-019-6290-0 PMC6829972

[52] McMahon M, Hatton C, Bowring DL: Polypharmacy and psychotropic polypharmacy in adults with intellectual disability: a cross-sectional total population study. *J Intellect Disabil Res* 2020. 10.1111/jir.12775 32902029

[53] Guan Q, Men S, Lunsky Y, Juurlink DN, Bronskill SE, Wunsch H, Gomes T. New opioid use and risk of opioid-related adverse events among adults with intellectual and developmental disabilities in Ontario, Canada. BJPsych Open. 2022;8(6): e208. 36440532 10.1192/bjo.2022.612 PMC9707500

[54] Kerins G, Petrovic K, Bruder MB, Gruman C. Medical conditions and medication use in adults with Down syndrome: a descriptive analysis. Downs Syndr Res Pract. 2008;12(2):141–7. 19026287 10.3104/reports.2009

[55] Carfì A, Romano A, Zaccaria G, Villani ER, Manes Gravina E, Vetrano DL, Bernabei R, Onder G. The burden of chronic disease, multimorbidity, and polypharmacy in adults with Down syndrome. Am J Med Genet A. 2020;182(7):1735–43. 32449279 10.1002/ajmg.a.61636

[56] de Knegt NC, Evenhuis HM, Lobbezoo F, Schuengel C, Scherder EJ. Does format matter for comprehension of a facial affective scale and a numeric scale for pain by adults with Down syndrome? Res Dev Disabil. 2013;34(10):3442–8. 23920027 10.1016/j.ridd.2013.07.016

[57] Sinnema M, Maaskant MA. van Schrojenstein Lantman-de Valk HMJ, Boer H, Curfs LMG, Schrander-Stumpel CTRM: The use of medical care and the prevalence of serious illness in an adult Prader-Willi syndrome cohort. Eur J Med Genet. 2013;56(8):397–403. 23792791 10.1016/j.ejmg.2013.05.011

[58] de Knegt NC, Lobbezoo F, Schuengel C, Evenhuis HM, Scherder EJA. Pain and Cognitive Functioning in Adults with Down Syndrome. Pain Med. 2017;18(7):1264–77. 28034975 10.1093/pm/pnw280

[59] Rousseau M-C, de Villemeur TB, Khaldi-Cherif S, Brisse C, Felce A, Loundou A, Baumstarck K, Auquier P, Leroy T, Haddadou S, et al. Polyhandicap and aging. Disabil Health J. 2019;12(4):657–64. 30842063 10.1016/j.dhjo.2019.01.013

[60] Bernal-Celestino RJ, León-Martín A, Martín-López MM, Ruiz-García J, Muñoz-Romera S, Lozano-Diaz AI. Evaluating and Handling the Pain of People With Intellectual Disability. Pain Manag Nurs. 2022;23(3):311–7. 34493439 10.1016/j.pmn.2021.08.005

[61] Pickering G, Boyer A, Danglades N, Arondo S, Lucchini C, Goubayon J, Dangin M, Boirie Y. Pain management in persons with intellectual disabilities living in institutions. Douleur et Analgesie. 2020;33(1):35–9.

[62] Holmes C, Brock K, Morgan P. Pain and its relationship with postural asymmetry in adults with cerebral palsy: A preliminary exploratory study. Disabil Health J. 2021;14(3): 101063. 33509734 10.1016/j.dhjo.2021.101063

[63] Findlay L. Williams ACdC, Scior K: Exploring experiences and understandings of pain in adults with intellectual disabilities. J Intellect Disabil Res. 2014;58(4):358–67. 23356659 10.1111/jir.12020

[64] Findlay L, Williams AC, Baum S, Scior K. Caregiver experiences of supporting adults with intellectual disabilities in pain. J Appl Res Intellect Disabil. 2015;28(2):111–20. 24909927 10.1111/jar.12109

[65] Nieuwenhuijse AM, Willems DL, van Goudoever JB, Olsman E. The perspectives of professional caregivers on quality of life of persons with profound intellectual and multiple disabilities: a qualitative study. Int J Dev Disabil. 2022;68(2):190–7. 35309693 10.1080/20473869.2020.1737469 PMC8928810

[66] Drozd M, Chadwick D, Jester R. The voices of people with an intellectual disability and a carer about orthopaedic and trauma hospital care in the UK: An interpretative phenomenological study. International Journal of Orthopaedic and Trauma Nursing. 2021;42: 100831. 33563567 10.1016/j.ijotn.2020.100831

[67] Pickering GL, Boyer A, Danglades N, Arondo S, Lucchini C, Goubayon J, Dangin M, Boirie Y. Pain management in persons with intellectual disabilities living in institutions. Douleur Et Analgesie. 2020;33:35–9.

[68] de Knegt NC, Lobbezoo F, Schuengel C, Evenhuis HM, Scherder EJA. Self-Reported Presence and Experience of Pain in Adults with Down Syndrome. Pain Med. 2017;18(7):1247–63. 27694149 10.1093/pm/pnw226

[69] Axmon A, Ahlström G, Gagnemo Persson R, Eberhard J. Demographic and diagnostic profiles of older people with intellectual disability and prescription of antipsychotics. Soc Psychiatry Psychiatr Epidemiol. 2019;54(8):937–44. 30903237 10.1007/s00127-019-01695-w

[70] The Anatomical Therapeutic Chemical (ATC) Classification System https://www.who.int/tools/atc-ddd-toolkit/atc-classification Accessed 3rd March 2024

[71] Lake JK, Balogh R, Lunsky Y. Polypharmacy profiles and predictors among adults with autism spectrum disorders. Research in Autism Spectrum Disorders. 2012;6(3):1142–9.

[72] Mojtabai R, Olfson M. National Trends in Psychotropic Medication Polypharmacy in Office-Based Psychiatry. Arch Gen Psychiatry. 2010;67(1):26–36. 20048220 10.1001/archgenpsychiatry.2009.175

[73] Laugharne R, Wilcock M, Rees J, Wainwright D, Newton N, Sterritt J, Badger S, Bishop R, Bassett P, Shankar R: Clinical characteristics of people with intellectual disability admitted to hospital with constipation: identifying possible specific high-risk factors. *Journal of Intellectual Disability Research*, n/a(n/a). 10.1111/jir.13108 38031737

[74] Harirforoosh S, Asghar W, Jamali F. Adverse Effects of Nonsteroidal Antiinflammatory Drugs: An Update of Gastrointestinal, Cardiovascular and Renal Complications. J Pharm Pharm Sci. 2014;16(5):821–47. 10.18433/j3vw2f 24393558

[75] van Timmeren EA, van der Schans CP, van der Putten AA, Krijnen WP, Steenbergen HA. van Schrojenstein Lantman-de Valk HM, Waninge A: Physical health issues in adults with severe or profound intellectual and motor disabilities: a systematic review of cross-sectional studies. J Intellect Disabil Res. 2017;61(1):30–49. 27228900 10.1111/jir.12296

[76] Amor-Salamanca A, Menchon JM. Pain underreporting associated with profound intellectual disability in emergency departments. J Intellect Disabil Res. 2017;61(4):341–7. 28054733 10.1111/jir.12355

[77] Hill S, Goldsmith L: Mobility, Posture and Comfort. In: *Profound Intellectual and Multiple Disabilities: Nursing Complex Needs.* edn.; 2009: 328–347.

[78] van der Putten A, Vlaskamp C. Pain assessment in people with profound intellectual and multiple disabilities; a pilot study into the use of the Pain Behaviour Checklist in everyday practice. Res Dev Disabil. 2011;32(5):1677–84. 21440413 10.1016/j.ridd.2011.02.020

[79] Disorders CtEtSSIDPfCwM: Mental Disorders and Disabilities Among Low-Income Children**.** In: *Clinical Characteristics of Intellectual Disabilities.* edn. Edited by Boat TF WJ: https://www.ncbi.nlm.nih.gov/books/NBK332877/; 2015.

[80] Robertson J, Baines S, Emerson E, Hatton C. Postural care for people with intellectual disabilities and severely impaired motor function: A scoping review. J Appl Res Intellect Disabil. 2018;31(S1):11–28. 28004472 10.1111/jar.12325

[81] Capone GT, Chicoine B, Bulova P, Stephens M, Hart S, Crissman B, Videlefsky A, Myers K, Roizen N, Esbensen A, et al. Co-occurring medical conditions in adults with Down syndrome: A systematic review toward the development of health care guidelines. Am J Med Genet A. 2018;176(1):116–33. 29130597 10.1002/ajmg.a.38512

[82] Coppus AM. People with intellectual disability: what do we know about adulthood and life expectancy? Dev Disabil Res Rev. 2013;18(1):6–16. 23949824 10.1002/ddrr.1123

[83] Boechler M, Fu YP, Raja N, Ruiz-Escobar E, Nimmagadda L, Osgood S, Levin MD, Hadigan C, Kozel BA: Gastrointestinal manifestations in Williams syndrome: A prospective analysis of an adult and pediatric cohort. *Am J Med Genet A* 2024:e63827. 10.1002/ajmg.a.63827 PMC11540717 39073239

[84] García-Domínguez L, Navas P, Verdugo M, Arias VB: Chronic Health Conditions in Aging Individuals with Intellectual Disabilities. *Int J Environ Res Public Health* 2020, **17**(9). 10.3390/ijerph17093126 PMC7246565 32365862

[85] Carey IM, Shah SM, Hosking FJ, DeWilde S, Harris T, Beighton C, Cook DG. Health characteristics and consultation patterns of people with intellectual disability: a cross-sectional database study in English general practice. Br J Gen Pract. 2016;66(645):e264–70. 26906630 10.3399/bjgp16X684301 PMC4809710

[86] Ho JSY, Collins G, Rohra V, Korb L, Perera B. Statin prescription and CV risk assessment in adult psychiatric outpatients with intellectual disability. Br J Cardiol. 2021;28(4):49. 35747067 10.5837/bjc.2021.049 PMC9063702

[87] CVD risk assessment and management.

[88] Weise J, Pollack A, Britt H, Trollor JN. Primary health care for people with an intellectual disability: an exploration of consultations, problems identified, and their management in Australia. J Intellect Disabil Res. 2017;61(5):399–410. 28116807 10.1111/jir.12352

[89] Ho IS, Azcoaga-Lorenzo A, Akbari A, Davies J, Hodgins P, Khunti K, Kadam U, Lyons R, McCowan C, Mercer SW, et al. Variation in the estimated prevalence of multimorbidity: systematic review and meta-analysis of 193 international studies. BMJ Open. 2022;12(4): e057017. 35487738 10.1136/bmjopen-2021-057017 PMC9058768

[90] Koponen MP, Bell JS, Karttunen NM, Nykänen IA, Desplenter FA, Hartikainen SA. Analgesic use and frailty among community-dwelling older people: a population-based study. Drugs Aging. 2013;30(2):129–36. 23288603 10.1007/s40266-012-0046-8

[91] Paulose-Ram R, Hirsch R, Dillon C, Losonczy K, Cooper M, Ostchega Y. Prescription and non-prescription analgesic use among the US adult population: results from the third National Health and Nutrition Examination Survey (NHANES III). Pharmacoepidemiol Drug Saf. 2003;12(4):315–26. 12812012 10.1002/pds.755

[92] Pokela N, Bell JS, Lihavainen K, Sulkava R, Hartikainen S. Analgesic use among community-dwelling people aged 75 years and older: A population-based interview study. Am J Geriatr Pharmacother. 2010;8(3):233–44. 20624613 10.1016/j.amjopharm.2010.05.001

[93] Tansuğ M, Kahraman T, Genç A. Differences in Pain Characteristics and Functional Associations between Nursing Home Residents and Community-Dwelling Older Adults: A Cross-Sectional Study. Ann Geriatr Med Res. 2021;25(3):187–96. 34433255 10.4235/agmr.21.0066 PMC8497941

[94] Bauer U, Pitzer S, Schreier MM, Osterbrink J, Alzner R, Iglseder B. Pain treatment for nursing home residents differs according to cognitive state – a cross-sectional study. BMC Geriatr. 2016;16(1):124. 27317390 10.1186/s12877-016-0295-1 PMC4912815

[95] Sheehan R, Hassiotis A, Walters K, Osborn D, Strydom A, Horsfall L. Mental illness, challenging behaviour, and psychotropic drug prescribing in people with intellectual disability: UK population based cohort study. BMJ. 2015;351: h4326. 26330451 10.1136/bmj.h4326 PMC4556752

[96] Prescribing of psychotropic drugs to people with learning disabilities and/or autism by general practitioners in England. Public Health England. (2015). [http://webarchive.nationalarchives.gov.uk/20160704152031/https://www.improvinghealthandlives.org.uk/publications/1248/Prescribing_of_psychotropic_medication_for_people_with_learning_disabilities_and_autism].

[97] Cooper S-A, Smiley E, Jackson A, Finlayson J, Allan L, Mantry D, Morrison J. Adults with intellectual disabilities: prevalence, incidence and remission of aggressive behaviour and related factors. J Intellect Disabil Res. 2009;53(3):217–32. 19178617 10.1111/j.1365-2788.2008.01127.x

[98] Emerson E, Kiernan C, Alborz A, Reeves D, Mason H, Swarbrick R, Mason L, Hatton C. The prevalence of challenging behaviors: a total population study. Res Dev Disabil. 2001;22(1):77–93. 11263632 10.1016/s0891-4222(00)00061-5

[99] Carr EG, Owen-DeSchryver JS. Physical Illness, Pain, and Problem Behavior in Minimally Verbal People with Developmental Disabilities. J Autism Dev Disord. 2007;37(3):413–24. 16897378 10.1007/s10803-006-0176-0

[100] Chadehumbe MA. Headache in Individuals with Neurodevelopmental Disorders. Curr Pain Headache Rep. 2023;27(10):623–9. 37566221 10.1007/s11916-023-01153-y

[101] Brylewski J, Duggan L: Antipsychotic medication for challenging behaviour in people with learning disability. *Cochrane Database Syst Rev* 2004(3):Cd000377. 10.1002/14651858.CD000377.pub2 PMC12334078 15266428

[102] van Schrojenstein Lantman-de Valk HM,. Walsh PN: Managing health problems in people with intellectual disabilities. BMJ. 2008;337: a2507. 19064601 10.1136/bmj.a2507

[103] Pergolizzi JV, LeQuang JA. Reappraising the use of benzodiazepines in chronic pain patients. Postgrad Med. 2020;132(sup3):10–2. 32008385 10.1080/00325481.2020.1725352

[104] Giovanni EF, Christina A-S, Martin U, Nanna BF, Richard OD, Andrew M, Sam E, Joshua RZ, Christopher GM. Efficacy, safety, and tolerability of antidepressants for pain in adults: overview of systematic reviews. BMJ. 2023;380: e072415. 36725015 10.1136/bmj-2022-072415 PMC9887507

[105] Wiffen PJ, Derry S, Moore RA, Aldington D, Cole P, Rice AS, Lunn MP, Hamunen K, Haanpaa M, Kalso EA: Antiepileptic drugs for neuropathic pain and fibromyalgia - an overview of Cochrane reviews. *Cochrane Database Syst Rev* 2013, 2013(11):Cd010567. 10.1002/14651858.CD010567.pub2 PMC6469538 24217986

[106] Boerlage AA, Valkenburg AJ, Scherder EJ, Steenhof G, Effing P, Tibboel D, van Dijk M. Prevalence of pain in institutionalized adults with intellectual disabilities: a cross-sectional approach. Res Dev Disabil. 2013;34(8):2399–406. 23714716 10.1016/j.ridd.2013.04.011

[107] Lonchampt S, Gerber F, Aubry JM, Desmeules J, Kosel M, Besson M. Prevalence of Polypharmacy and Inappropriate Medication in Adults With Intellectual Disabilities in a Hospital Setting in Switzerland. Front Psychiatry. 2021;12: 614825. 34248693 10.3389/fpsyt.2021.614825 PMC8267250

[108] Declaration of Montreal- Access to Pain Management Is a Fundamental Human Right- Last updated: September 15, 2015.https://www.iasp-pain.org/advocacy/iasp-statements/access-to-pain-management-declaration-of-montreal/ Accessed 9th March 2024 [https://www.iasp-pain.org/advocacy/iasp-statements/access-to-pain-management-declaration-of-montreal/].

[109] Gréaux M, Moro MF, Kamenov K, Russell AM, Barrett D, Cieza A. Health equity for persons with disabilities: a global scoping review on barriers and interventions in healthcare services. International Journal for Equity in Health. 2023;22(1):236. 37957602 10.1186/s12939-023-02035-w PMC10644565

[110] Maulik PK, Mascarenhas MN, Mathers CD, Dua T, Saxena S. Prevalence of intellectual disability: A meta-analysis of population-based studies. Res Dev Disabil. 2011;32(2):419–36. 21236634 10.1016/j.ridd.2010.12.018

