# Supplementary material for: Investigating pain-related medication use and contribution to polypharmacy in adults with intellectual disabilities: a systematic review
Source: BMC Med. 2024 Dec 2;22:565. doi: 10.1186/s12916-024-03770-9 (PMC11610167; doi:10.1186/s12916-024-03770-9)
Supplement: Supplementary file 1 — Additional file 1. Search terms examples. [file 12916_2024_3770_MOESM1_ESM.docx]

**ADDITIONAL FILE 1-SEARCH TERMS EXAMPLE**

**OVID (Medline, Web of Science, Embase)**

1. (tuberous sclerosis or de lange or phenylketonuria or mucopolysaccharidosis or Fragile X or Prader Willi or Williams Beuren or neural tube defect or congenital rubella syndrome or fetal alcohol syndrome or trisomy or cerebral palsy or angelman or happy puppet or otahara or lennox gastaut or sturge weber or infantile spasm or west syndrome or autis*).mp. [mp=title, abstract, heading word, drug trade name, original title, device manufacturer, drug manufacturer, device trade name, keyword heading word, floating subheading word, candidate term word
2. exp intellectual impairment/
3. ((intellectual* or learning or neurodevelopment*) adj3 (handicap* or retard* or disab* or difficult* or impair* or deficienc* or incapacit* or delay* or problem* or condition* or disturb or subaverage or disorder)).mp. [mp=title, abstract, heading word, drug trade name, original title, device manufacturer, drug manufacturer, device trade name, keyword heading word, floating subheading word, candidate term word]
4. (special needs or Down Syndrome or learning disorder).mp. [mp=title, abstract, heading word, drug trade name, original title, device manufacturer, drug manufacturer, device trade name, keyword heading word, floating subheading word, candidate term word]
5. 1 or 2 or 3 or 4
6. exp pain/
7. exp drug therapy/
8. exp opiate/ or exp analgesia/ or exp analgesic agent/
9. 7 or 8
10. 5 and 6 and 9
11. exp human/
12. 10 and 11
13. exp adult/ or exp young adult/ or exp institutionalized adult/ or exp adult child/ or exp transition to adult care/
14. 12 and 13
15. limit 14 to yr="2000 -Current"

**EBSCO (CINAHL, APA Psycinfo)**

1. TI ( ("tuberous sclerosis" or "de lange" or phenylketonuria or mucopolysaccharidosis or "Fragile X" or "Prader Willi" or "Williams Beuren" or "neural tube defect" or "congenital rubella syndrome" or "fe$tal alcohol syndrome" or trisomy or "cerebral palsy" or angelman or "happy puppet" or otahara or "lennox gastaut" or "sturge weber" or "infantile spasm" or "west syndrome" or autis*) ) OR AB ( ("tuberous sclerosis" or "de lange" or phenylketonuria or mucopolysaccharidosis or "Fragile X" or "Prader Willi" or "Williams Beuren" or "neural tube defect" or "congenital rubella syndrome" or "fe$tal alcohol syndrome" or trisomy or "cerebral palsy" or angelman or "happy puppet" or otahara or "lennox gastaut" or "sturge weber" or "infantile spasm" or "west syndrome" or autis*) )
2. TI intellectual impairment OR AB intellectual impairment
3. TI ( "intellectual* or learning or neurodevelopment*" n3 "handicap* or retard* or disab* or difficult* or impair* or deficienc* or incapacit* or delay* or problem* or condition* or disturb* or subaverage* or disorder* ) OR AB ( "intellectual* or learning or neurodevelopment*" n3 "handicap* or retard* or disab* or difficult* or impair* or deficienc* or incapacit* or delay* or problem* or condition* or disturb* or subaverage* or disorder* )
4. TI ( "special needs" or "Down Syndrome" or "learning disorder" ) OR AB ( "special needs" or "Down Syndrome" or "learning disorder" )
5. S1 OR S2 OR S3 OR S4
6. TI pain OR AB pain
7. TI drug therapy OR AB drug therapy
8. TI ( opiate or analgesi* or "analgesic agent" ) OR AB ( opiate or analgesi* or "analgesic agent" )
9. S7 OR S8
10. S5 AND S6 AND S9
11. S10 AND S11

**PUBMED**

("tuberous sclerosis"[MeSH Terms] OR ("tuberous"[All Fields] AND "sclerosis"[All Fields]) OR "tuberous sclerosis"[All Fields] OR (lange, de[Author] OR de lange[Author] OR de lange[Investigator]) OR ("phenylketonurias"[MeSH Terms] OR "phenylketonurias"[All Fields] OR "phenylketonuria"[All Fields]) OR ("mucopolysaccharidose"[All Fields] OR "mucopolysaccharidoses"[MeSH Terms] OR "mucopolysaccharidoses"[All Fields] OR "mucopolysaccharidosis"[All Fields]) OR ("Fragile"[All Fields] AND "X"[All Fields]) OR (("prader"[All Fields] OR "prader s"[All Fields]) AND "Willi"[All Fields]) OR (("william"[All Fields] OR "williams"[All Fields] OR "williams s"[All Fields]) AND ("beuren"[All Fields] OR "beuren s"[All Fields])) OR ("neural tube defects"[MeSH Terms] OR ("neural"[All Fields] AND "tube"[All Fields] AND "defects"[All Fields]) OR "neural tube defects"[All Fields] OR ("neural"[All Fields] AND "tube"[All Fields] AND "defect"[All Fields]) OR "neural tube defect"[All Fields]) OR ("rubella syndrome, congenital"[MeSH Terms] OR ("rubella"[All Fields] AND "syndrome"[All Fields] AND "congenital"[All Fields]) OR "congenital rubella syndrome"[All Fields] OR ("congenital"[All Fields] AND "rubella"[All Fields] AND "syndrome"[All Fields])) OR ("foetal alcohol syndrome"[All Fields] OR "fetal alcohol spectrum disorders"[MeSH Terms] OR ("fetal"[All Fields] AND "alcohol"[All Fields] AND "spectrum"[All Fields] AND "disorders"[All Fields]) OR "fetal alcohol spectrum disorders"[All Fields] OR ("fetal"[All Fields] AND "alcohol"[All Fields] AND "syndrome"[All Fields]) OR "fetal alcohol syndrome"[All Fields]) OR ("trisomie"[All Fields] OR "trisomy"[MeSH Terms] OR "trisomy"[All Fields] OR "trisomies"[All Fields]) OR ("cerebral palsy"[MeSH Terms] OR ("cerebral"[All Fields] AND "palsy"[All Fields]) OR "cerebral palsy"[All Fields]) OR ("angelman"[All Fields] OR "angelman s"[All Fields]) OR (("happi"[All Fields] OR "happiness"[MeSH Terms] OR "happiness"[All Fields] OR "happy"[All Fields]) AND ("play and playthings"[MeSH Terms] OR ("play"[All Fields] AND "playthings"[All Fields]) OR "play and playthings"[All Fields] OR "puppet"[All Fields] OR "puppets"[All Fields] OR "puppet s"[All Fields] OR "puppeteer"[All Fields])) OR "otahara"[All Fields] OR ("lennox"[All Fields] AND "gastaut"[All Fields]) OR ("sturge"[All Fields] AND ("weber"[All Fields] OR "webers"[All Fields])) OR ("spasms, infantile"[MeSH Terms] OR ("spasms"[All Fields] AND "infantile"[All Fields]) OR "infantile spasms"[All Fields] OR ("infantile"[All Fields] AND "spasm"[All Fields]) OR "infantile spasm"[All Fields]) OR ("spasms, infantile"[MeSH Terms] OR ("spasms"[All Fields] AND "infantile"[All Fields]) OR "infantile spasms"[All Fields] OR ("west"[All Fields] AND "syndrome"[All Fields]) OR "west syndrome"[All Fields]) OR "autis*"[All Fields]) AND ((humans[Filter]) AND (alladult[Filter])) ("intellectual disability"[MeSH Terms]) AND ((humans[Filter]) AND (alladult[Filter])) ("intellectual*"[All Fields] OR "learning"[MeSH Terms] OR "learning"[All Fields] OR "learn"[All Fields] OR "learned"[All Fields] OR "learning s"[All Fields] OR "learnings"[All Fields] OR "learns"[All Fields] OR "neurodevelopment*"[All Fields] OR "handicap*"[All Fields] OR "retard*"[All Fields] OR "disab*"[All Fields] OR "difficult*"[All Fields] OR "impair*"[All Fields] OR "deficienc*"[All Fields] OR "incapacit*"[All Fields] OR "delay*"[All Fields] OR "problem*"[All Fields] OR "condition*"[All Fields] OR "disturb"[All Fields] OR "disturbance"[All Fields] OR "disturbances"[All Fields] OR "disturbancies"[All Fields] OR "disturbed"[All Fields] OR "disturbing"[All Fields] OR "disturbs"[All Fields] OR "subaverage"[All Fields] OR "subaveraged"[All Fields] OR "subaverages"[All Fields] OR "disease"[MeSH Terms] OR "disease"[All Fields] OR "disorder"[All Fields] OR "disorders"[All Fields] OR "disorder s"[All Fields] OR "disordes"[All Fields]) AND ((humans[Filter]) AND (alladult[Filter])) ((("medicine"[MeSH Terms] OR "medicine"[All Fields] OR "specialty"[All Fields] OR "special"[All Fields] OR "specialisations"[All Fields] OR "specialise"[All Fields] OR "specialised"[All Fields] OR "specialises"[All Fields] OR "specialising"[All Fields] OR "specialisms"[All Fields] OR "specialities"[All Fields] OR "speciality"[All Fields] OR "specialization"[MeSH Terms] OR "specialization"[All Fields] OR "specialisation"[All Fields] OR "specialism"[All Fields] OR "specializations"[All Fields] OR "specialize"[All Fields] OR "specialized"[All Fields] OR "specializes"[All Fields] OR "specializing"[All Fields] OR "specially"[All Fields] OR "specials"[All Fields] OR "specialties"[All Fields] OR "specialty s"[All Fields]) AND ("health services needs and demand"[MeSH Terms] OR ("health"[All Fields] AND "services"[All Fields] AND "needs"[All Fields] AND "demand"[All Fields]) OR "health services needs and demand"[All Fields] OR "needed"[All Fields] OR "needs"[All Fields] OR "needing"[All Fields])) OR ("down syndrome"[MeSH Terms] OR ("down"[All Fields] AND "syndrome"[All Fields]) OR "down syndrome"[All Fields]) OR ("learning disabilities"[MeSH Terms] OR ("learning"[All Fields] AND "disabilities"[All Fields]) OR "learning disabilities"[All Fields] OR ("learning"[All Fields] AND "disorder"[All Fields]) OR "learning disorder"[All Fields])) AND ((humans[Filter]) AND (alladult[Filter])) ("pain"[MeSH Terms]) AND ((humans[Filter]) AND (alladult[Filter])) ("drug therapy"[MeSH Terms]) AND ((humans[Filter]) AND (alladult[Filter])) ("opiate alkaloids"[MeSH Terms] OR ("opiate"[All Fields] AND "alkaloids"[All Fields]) OR "opiate alkaloids"[All Fields] OR "opiate"[All Fields] OR "opiates"[All Fields] OR "opiate s"[All Fields] OR ("analgesia"[MeSH Terms] OR "analgesia"[All Fields] OR "analgesias"[All Fields]) OR "analgesics"[MeSH Terms]) AND ((humans[Filter]) AND (alladult[Filter]))
