# Supplementary material for: Investigating pain-related medication use and contribution to polypharmacy in adults with intellectual disabilities: a systematic review
Source: BMC Med. 2024 Dec 2;22:565. doi: 10.1186/s12916-024-03770-9 (PMC11610167; doi:10.1186/s12916-024-03770-9)
Supplement: Supplementary file 2 — Additional file 2. Quality assessment of studies. [file 12916_2024_3770_MOESM2_ESM.docx]

**Additional File 2- Quality Appraisal of Included Articles.**

Critical Appraisal Skills Programme (CASP) for Qualitative Studies

| **Study** | **Q1** | **Q2** | **Q3** | **Q4** | **Q5** | **Q6** | **Q7** | **Q8** | **Q9** | **Q10** | **Score** |
| --- | --- | --- | --- | --- | --- | --- | --- | --- | --- | --- | --- |
| Findlay et. al. (2013) - | Yes | Yes | Yes | Yes | Yes | Can’t tell | Yes | Yes | Yes | Yes | **19/20** |
| Findlay et. al. (2014) | Yes | Yes | Yes | Yes | Yes | Can’t tell | Yes | Yes | Yes | Yes | **19/20** |
| Drozd et. al. (2021) | Yes | Yes | Yes | Yes | Yes | No | Yes | Yes | Yes | Yes | **18/20** |
| Nieuwenhuijse et. al. (2022) | Yes | Yes | Yes | Yes | Yes | No | Yes | Yes | Yes | Yes | **18/20** |

***Critical Appraisal Skills Programme(CASP) questions scoring : Yes= 2, Can’t tell= 1, No =0***

Q1. Was there a clear statement of the aims of the research?

Q2. Is a qualitative methodology appropriate?

Q3. Was the research design appropriate to address the aims of the research?

Q4. Was the recruitment strategy appropriate to the aims of the research?

Q5. Was the data collected in a way that addressed the research issue?

Q6. Has the relationship between researcher and participants been adequately considered?

Q7. Have ethical issues been taken into consideration?

Q8. Was the data analysis sufficiently rigorous?

Q9. Is there a clear statement of findings?

Q10. How valuable is the research?

Adapted Newcastle-Ottawa Scale for Observational Studies

| **Reference** | Kerins et al (2008) | Walsh et al (2011) | Turk et al (2011) | De Knegt et al (2013) | Sinnema et al  (2013) | Doan et al  (2013) | | Cocks et al (2016) | O’Dwyer et al  (2016) | Bowring et al  (2017) | Peklar et al  (2017) |
| --- | --- | --- | --- | --- | --- | --- | --- | --- | --- | --- | --- |
| **SELECTION.** | | | | | | | | | | | |
| 1. Representative of adults with ID | | | | | | | | | | | |
| a) Truly representative* | **X** | **X** |  | **X** | **X** | **X** | |  | **X** | **X** | **X** |
| b) Somewhat representative* |  |  | **X** |  |  |  | | **X** |  |  |  |
| c) Selected group |  |  |  |  |  |  | |  |  |  |  |
| d) No description of the derivation of the study population |  |  |  |  |  |  | |  |  |  |  |
| 2. Ascertainment of confirmation of ID | | | | | | | | | | | |
| a) Secure record of method of identification of AWID (administrative health or social care dataset, medical record, validated assessment)* | **X** |  |  |  |  |  | |  | **X** | **X** | **X** |
| b) Identified by means of residential care or support delivered by an organisation or caregivers of AWID, or caregiver report* |  |  |  |  |  |  | |  |  |  |  |
| c) Mix of both* |  | **X** | **X** | **X** | **X** | **X** | | **X** |  |  |  |
| d) No description of means of identification of AWID |  |  |  |  |  |  | |  |  |  |  |
| 3. Non-respondents | **N/A** |  |  |  |  | |  |  | **N/A** | **N/A** | **N/A** |
| a) Comparability between respondents & non-respondents characteristics is established, & response rate satisfactory* |  |  | **X** | **X** | **X** | **X** | | **X** |  |  |  |
| b) Response rate unsatisfactory, or comparability between respondents & non-respondents unsatisfactory |  | **X** |  |  |  |  | |  |  |  |  |
| c) No description of response rate or characteristics of responders and non-responders |  |  |  |  |  |  | |  |  |  |  |
| **(3 stars- strong; 2 stars-moderate; 0/1 stars-weak)** | **STRONG**  **(2/2)** | **MODERATE**  **(2/3)** | **STRONG**  **(3/3)** | **STRONG**  **(3/3)** | **STRONG**  **(3/3)** | **STRONG**  **(3/3)** | | **STRONG**  **(3/3)** | **STRONG**  **(2/2)** | **MODERATE**  **(2/2)** | **STRONG**  **(2/2)** |
| **COMPARABILITY** | | | | | | | | | | | |
| 4. Key potential confounders measured & controlled (max two) where comparator sample included in study | **N/A** | **N/A** |  |  | **N/A** | **N/A** | |  | **N/A** | **N/A** |  |
| a) Study controls for age, sex, ID severity & underlying cause of ID* |  |  |  | **X** |  |  | | **X** |  |  | **X** |
| b) Study controls for other factors: e.g. education level, BMI, comorbid diagnosis* |  |  | **X** |  |  |  | | **X** |  |  |  |
| c) Sub-groups are not comparable on basis of design or analysis not controlled for confounders or no description of sub-groups |  |  |  |  |  |  | |  |  |  |  |
| **Rate the section (1/2 stars-strong; 0 star-weak)** | **N/A** | **N/A** | **STRONG** | **STRONG** | **N/A** | **N/A** | | **STRONG** | **N/A** | **N/A** | **STRONG** |
| **OUTCOME & DISCUSSION** | | | | | | | | | | | |
| 5. Assessment of outcomes related to pain medication, multimorbidity and polypharmacy |  |  |  |  |  |  | |  |  |  |  |
| a) Electronic Health Records* | **X** | **X** |  |  |  |  | |  | **X** |  |  |
| b) Carer or self-report* |  |  | **X** |  |  | **X** | |  |  |  |  |
| c) Mix of both* |  |  |  | **X** | **X** |  | | **X** |  | **X** | **X** |
| d) Not described |  |  |  |  |  |  | |  |  |  |  |
| 6. Were the limitation of the study discussed? |  |  |  |  |  |  | |  |  |  |  |
| a) Yes* | **X** | **X** |  | **X** |  | **X** | | **X** | **X** | **X** | **X** |
| b) No |  |  | **X** |  | **X** |  | |  |  |  |  |
| 7. Were there any conflicts of interest declared? |  |  |  |  |  |  | |  |  |  |  |
| a) No conflicts of interested* |  | **X** |  | **X** |  |  | |  |  |  |  |
| b) Funding explained, no effect on study results* |  |  | **X** |  |  | **X** | | **X** | **X** |  | **X** |
| c) Competing interests exist |  |  |  |  |  |  | |  |  |  |  |
| d) No statement | **X** |  |  |  | **X** |  | |  |  | **X** |  |
| **Rate the section (3 stars-strong, 2 stars -moderate 0/1 star-weak)** | **MODERATE**  **(2/3)** | **STRONG**  **(3/3)** | **MODERATE**  **(3/3)** | **STRONG**  **(3/3)** | **WEAK**  **(1/3)** | **STRONG**  **(3/3)** | | **STRONG**  **(3/3)** | **STRONG**  **(3/3)** | **MODERATE**  **(2/3)** | **STRONG**  **(3/3)** |
| **Overall rating (All strong-good; at least one weak-poor; rest-fair)** | **FAIR** | **FAIR** | **FAIR** | **GOOD** | **POOR** | **GOOD** | | **GOOD** | **GOOD** | **FAIR** | **GOOD** |

Adapted Newcastle-Ottawa Scale for Observational Studies.(cont.)

| **Reference** | De Knegt et al  (2017) | Axmon et al  (2017) | Salomon et al  (2018) | Axmon et al  (2018) | Hove et al  (2018) | Carfi et al  (2019) | Rosseau et al  (2019) | Segerlanz et al  (2019) | Pickering et al  (2020) |
| --- | --- | --- | --- | --- | --- | --- | --- | --- | --- |
| **SELECTION.** | | | | | | | | | |
| 1. Representative of adults with ID | | | | | | | | | |
| a) Truly representative* |  |  |  |  | **X** |  | **X** |  | **X** |
| b) Somewhat representative* | **X** | **X** | **X** | **X** |  | **X** |  | **X** |  |
| c) Selected group |  |  |  |  |  |  |  |  |  |
| d) No description of the derivation of the study population |  |  |  |  |  |  |  |  |  |
| 2. Ascertainment of confirmation of ID | | | | | | | | | |
| a) Secure record of method of identification of AWID (administrative health or social care dataset, medical record, validated assessment)* |  | **X** | **X** | **X** |  | **X** | **X** | **X** | **X** |
| b) Identified by means of residential care or support delivered by an organisation or caregivers of AWID, or caregiver report* |  |  |  |  |  |  |  |  |  |
| c) Mix of both* | **X** |  |  |  | **X** |  |  |  |  |
| d) No description of means of identification of AWID |  |  |  |  |  |  |  |  |  |
| 3. Non-respondents |  | **N/A** | **N/A** | **N/A** |  | **N/A** | **N/A** | **N/A** | **N/A** |
| a) Comparability between respondents & non-respondents characteristics is established, & response rate satisfactory* |  |  |  |  |  |  |  |  |  |
| b) Response rate unsatisfactory, or comparability between respondents & non-respondents unsatisfactory |  |  |  |  |  |  |  |  |  |
| c) No description of response rate or characteristics of responders and non-responders | **X** |  |  |  | **X** |  |  |  |  |
| **(3/3 or2/2 stars- strong; 2/3 or 1/2 stars-moderate; 0/1 stars-weak)** | **MODERATE**  **(2/3)** | **STRONG**  **(2/2)** | **STRONG**  **(2/2)** | **STRONG**  **(2/2)** | **MODERATE**  **(2/3)** | **STRONG**  **(2/2)** | **STRONG**  **(2/2)** | **STRONG**  **(2/2)** | **STRONG**  **(2/2)** |
| **COMPARABILITY** | | | | | | | | | |
| 4. Key potential confounders measured & controlled (max two) where comparator population included in study | **N/A** |  |  |  |  | **N/A** | **N/A** |  | **N/A** |
| a) Study controls for age, sex, ID severity & underlying cause of ID* |  |  |  |  |  |  |  |  |  |
| b) Study controls for other factors: e.g. education level, BMI, comorbid diagnosis* |  |  |  |  |  |  |  |  |  |
| c) Sub-groups are not comparable on basis of design or analysis not controlled for confounders or no description of sub-groups |  | **X** | **X** | **X** | **X** |  |  | **X** |  |
| **Rate the section (1/2 stars-strong; 0 star-weak)** | **N/A** | **WEAK** | **WEAK** | **WEAK** | **WEAK** | **N/A** | **N/A** | **WEAK** | **N/A** |
| **OUTCOME & DISCUSSION** | | | | | | | | | |
| 5. Assessment of outcomes related to pain medication, multimorbidity and polypharmacy. |  |  |  |  |  |  |  |  |  |
| a) Electronic Health Records* |  | **X** | **X** | **X** |  | **X** | **X** | **X** |  |
| b) Carer or self-report* |  |  |  |  |  |  |  |  |  |
| c) Mix of both* | **X** |  |  |  | **X** |  |  |  | **X** |
| d) Not described |  |  |  |  |  |  |  |  |  |
| 6. Were the limitation of the study discussed? |  |  |  |  |  |  |  |  |  |
| a) Yes* | **X** | **X** | **X** | **X** | **X** | **X** | **X** | **X** |  |
| b) No |  |  |  |  |  |  |  |  | **X** |
| 7. Were there any conflicts of interest declared? |  |  |  |  |  |  |  |  |  |
| a) No conflicts of interest* |  | **X** |  | **X** | **X** |  | **X** | **X** | **X** |
| b) Funding explained, no effect on study results* |  |  |  |  |  |  |  |  |  |
| c) Competing interests exist |  |  | **X** |  |  |  |  |  |  |
| d) No statement | **X** |  |  |  |  | **X** |  |  |  |
| **Rate the section (3 stars-strong, 2 stars -moderate 0/1 star-weak)** | **MODERATE**  **(2/3)** | **STRONG**  **(3/3)** | **MODERATE**  **(2/3)** | **STRONG**  **(3/3)** | **STRONG**  **(3/3)** | **MODERATE**  **(2/3)** | **STRONG**  **(3/3)** | **STRONG**  **(3/3)** | **MODERATE**  **(2/3)** |
| **Overall rating (All strong-good; at least one weak-poor; rest-fair)** | **FAIR** | **POOR** | **POOR** | **POOR** | **POOR** | **FAIR** | **GOOD** | **POOR** | **FAIR** |

Adapted Newcastle-Ottawa Scale for Observational Studies.(cont.)

| **Reference** | McMahon et al  (2020) | Holmes et al (2021) | Guan et al(2022) | Bernal- Celestino et al (2022) |
| --- | --- | --- | --- | --- |
| **SELECTION.** | | | | |
| 1. Representative of adults with ID | | | | |
| a) Truly representative* | **X** |  |  | **X** |
| b) Somewhat representative* |  |  | **X** |  |
| c) Selected group |  | **X** |  |  |
| d) No description of the derivation of the study population |  |  |  |  |
| 2. Ascertainment of confirmation of ID | | | | |
| a) Secure record of method of identification of AWID (administrative health or social care dataset, medical record, validated assessment)* | **X** | **X** | **X** |  |
| b) Identified by means of residential care or support delivered by an organisation or caregivers of AWID, or caregiver report* |  |  |  |  |
| c) Mix of both* |  |  |  | **X** |
| d) No description of means of identification of AWID |  |  |  |  |
| 3. Non-respondents | **N/A** | **N/A** | **N/A** |  |
| a) Comparability between respondents & non-respondents characteristics is established, & response rate satisfactory* |  |  |  |  |
| b) Response rate unsatisfactory, or comparability between respondents & non-respondents unsatisfactory |  |  |  |  |
| c) No description of response rate or characteristics of responders and non-responders |  |  |  | **X** |
| **Rate the section (3/3, 2/2 stars- strong; 2/3, 1/2 stars-moderate; 0/1 stars-weak)** | **STRONG**  **(2/2)** | **MODERATE**  **(1/2)** | **STRONG**  **(2/2)** | **MODERATE**  **(2/3)** |
| **COMPARABILITY** | | | | |
| 4. Key potential confounders measured & controlled (max two) where comparator population included in study | **N/A** | **N/A** |  |  |
| a) Study controls for age, sex, ID severity & underlying cause of ID* |  |  |  |  |
| b) Study controls for other factors: e.g. education level, BMI, comorbid diagnosis* |  |  |  |  |
| c) Sub-groups are not comparable on basis of design or analysis not controlled for confounders or no description of sub-groups |  |  | **X** | **X** |
| **Rate the section (1/2 stars-strong; 0 star-weak)** | **N/A** | **N/A** | **WEAK** | **WEAK** |
| **OUTCOME & DISCUSSION** | | | | |
| 5. Assessment of outcomes related to pain medication, multimorbidity and polypharmacy |  |  |  |  |
| a) Electronic Health Records* | **X** |  | **X** |  |
| b) Carer or self- reports* |  |  |  |  |
| c) Mix of both* |  | **X** |  | **X** |
| d) Not described |  |  |  |  |
| 6. Were the limitation of the study discussed? |  |  |  |  |
| a) Yes* | **X** |  | **X** | **X** |
| b) No |  | **X** |  |  |
| 7. Were there any conflicts of interest declared? |  |  |  |  |
| a) No conflicts of interest* | **X** | **X** |  | **X** |
| b) Funding explained, no effect on study results* |  |  | **X** |  |
| c) Competing interests exist |  |  |  |  |
| d) No statement |  |  |  |  |
| **Rate the section (3 stars-strong, 2 stars -moderate 0/1 star-weak)** | **STRONG**  **(3/3)** | **MODERATE**  **(2/3)** | **STRONG**  **(3/3)** | **STRONG** |
| **Overall rating (All strong-good; at least one weak-poor; rest-fair)** | **GOOD** | **FAIR** | **POOR** | **POOR** |
